# Bacterial microbiota protect an invasive bark beetle from a pine defensive compound

**DOI:** 10.1186/s40168-018-0518-0

**Published:** 2018-07-27

**Authors:** Chihang Cheng, Jacob D Wickham, Li Chen, Dandan Xu, Min Lu, Jianghua Sun

**Affiliations:** 1 State Key Laboratory of Integrated Management of Pest Insects and Rodents, Institute of Zoology, Chinese Academy of Sciences, 1 Beichen West Road, Chaoyang District, Beijing, 100101 China; 2 University of the Chinese Academy of Sciences, Beijing, 100049 China; 3 College of Life Sciences, Huzhou University, No. 759, East 2nd Road, Huzhou, 313000 China

**Keywords:** Community structure, Biodegradation, Biological invasion, Symbiotic microbiota, Protection, Pyrosequencing

## Abstract

**Background:**

There is growing evidence that some devastating biotic invasions are facilitated by microbial symbionts. The red turpentine beetle (RTB), an innocuous secondary insect attacking weakened trees in North America, has formed an invasive complex with the fungus *Leptographium procerum* in China, and this invasive beetle-fungus symbiotic complex is capable of attacking and killing healthy pines. A previous study demonstrated that three Chinese-resident fungi, newly acquired by RTB in China, induce high levels of a phenolic defensive chemical, naringenin, in pines and this invasive beetle-fungus complex is suppressed by elevated levels of naringenin while the beetle uses its gallery as an external detoxification system in which particular yeast-like fungi and bacterial species biodegrade naringenin. However, the functional roles of key microbial players in the symbiosis, contained within the microbiome of the bark beetle gallery, have not been well elucidated.

**Results:**

In this report, the symbiotic naringenin-degrading microbiota were found to increase RTB survivorship in the presence of induced host defenses, and potential genes associated with degradation pathways were discovered. While fungi in the gallery microbiota had little involvement in naringenin degradation, bacterial community structure within the beetle gallery was highly correlated to naringenin degrading activity. Phylotypes of the Gram-negative bacterial genus *Novosphingobium*, which possessed genes involved in degradation pathways, were highly correlated to naringenin degradation activities and RTB associated with an isolated species of this genus acquired protection against naringenin and gained fitness.

**Conclusions:**

Our results demonstrated that symbiotic bacterial community of RTB galleries enhances the survivorship and overall fitness of invasive beetles by degrading the host phenolic naringenin, ultimately overcoming the tree defenses and facilitating the success of the invasive beetle-fungi complex. This dynamic interplay between the invasive insect pest and multipartite microbes suggests a putative mechanism in invasion ecology for mitigating biotic resistance to symbiotic invasion.

**Electronic supplementary material:**

The online version of this article (10.1186/s40168-018-0518-0) contains supplementary material, which is available to authorized users.

## Background

Invasive species change and disrupt global ecosystems [1, 2]. Among the many mechanisms underlying rapid adaptation and range expansion by invasive species, the positive feedback interactions with symbiotic microbes have been recognized to be of prevailing importance [3, 4]. The extended functional traits provided by symbiotic microbes contribute to the competitiveness and fitness of their invasive hosts. These traits, as part of extended phenotypes for some exotic plants and animals, have been attributed to particular symbiotic microbes [5, 6]. For example, the endophytic fungus *Neotyphodium coenophialum* in the invasive grass *Lolium arundinaceum* confers herbivore resistance for the host plant in its introduced range in the USA [7, 8]; fungal endophytes *Alternaria* spp. in the invasive forb *Centaurea stoebe* increase host competitive ability against North American native grasses [9]; an endosymbiotic bacterium in the genus *Rickettsia* improves reproduction in the invasive whitefly (*Bemisia tabaci*) [10]; and microsporidia in the invasive ladybird *Harmonia axyridis* are involved in killing off native competitors in Europe [11].

Microbes that function to increase fitness of invaders reside alongside microbes with unknown functions or those that opportunistically colonize a variety of environmental habitats [12, 13]. Rhizospheres and roots of exotic plants and the living nests, bodies, and guts of exotic insects are well-known microhabitats colonized by various bacterial and fungal members [14–18]. These co-occurring microbes compose symbiotic microbiota that provide benefits for invasive species [19–21]. The community structure, composition, and expression profiles, however, can change over time or in response to biotic and abiotic events, making it difficult to identify functional microbes without considering the dynamic interplay of microbes in the communities. Therefore, it is crucial to elucidate the key players of symbiotic microbiota that are involved in facilitating successful invasions, from the community level down to the particular taxon or taxa directly involved. While much work has been done to understand the roles of the key symbiotic microbiota, there are still many knowledge gaps.

The red turpentine beetle (RTB) *Dendroctonus valens* and *Leptographium procerum*, the fungus it vectors, were simultaneously introduced from North America to China and act as an invasive beetle-fungus complex that devastates healthy pine forests in its introduced range [22]. *L. procerum* is significantly pathogenic to *Pinus tabuliformis*, its host pine tree, and induces it to release high amounts of Δ-3-carene, a volatile terpenoid which is attractive for the beetle vector [23, 24]. During the invasion process, RTB has associated with Chinese-resident fungi which specifically induce a defensive phenolic compound naringenin, from the host pine. Naringenin strongly suppresses the invasive beetle-fungus complex [25]. Interestingly, the RTB gallery microenvironment (space between bark and tree wood) with its microbial community serves as an external detoxification system, where the multiple associated yeasts and bacteria help eliminate this harmful compound via biodegradation [25]. *L. procerum* maintains host pine carbohydrate pinitol for the growth of gallery microbiota and facilitates naringenin biodegradation by the microbiota, forming a mutualistic relationship between RTB, *L. procerum*, and gallery microbiota [25].

Although several species of yeasts and bacteria have been isolated and assessed for their naringenin biodegrading activities, community-level evaluation of naringenin biodegradation function of gallery microbiota is lacking. Thus, it is still unclear which key microbial players actively participate in this key function that could be responsible for the protection of the invasive beetle-fungus complex. In order to explore which microbes in RTB gallery microbiota play key roles in protecting the invasive beetle through naringenin biodegradation, we proposed the following hypotheses: first, whether symbiotic naringenin-degrading microbiota in the gallery can protect invasive RTB from naringenin; second, what microbial group(s), either bacteria and/or fungi, correlate(s) to naringenin-biodegrading activity of the gallery community.

## Methods

### Sample collection

We collected RTB gallery tissues from naturally infested *P. tabuliformis* trees at Tunlanchuan Forest Station (37° 48′ N, 111° 44′ E; average elevation 1400 m; *P. tabuliformis* 3278 ha) in Shanxi Province. Gallery samples (ca. 0.5 g in fresh weight) were first homogenized into fine pieces by sterile forceps and scissors and then macerated in 0.5 ml 10% PBS (phosphate-buffered saline) (Sigma) to obtain crude extracts containing microbial cells (gallery microbiota).

Second to third instar RTB larvae were randomly collected from beetle galleries of infested host pines at Tunlanchuan Forest Station. Beetles were surface-sterilized with bleach, ethanol, and distilled water [10:10:80 (vol:vol)], kept in a climate-controlled incubator (25 ± 1 °C, RH = 70%, darkness) and starved for 24 h before use.

### Beetle survivorship bioassay with gallery microbiota

Phloem media (phloem powder 8 g; bacto-agar 2 g; distilled water 60 ml) amended with naringenin (2000 μg g^−1^ of media dry weight) was prepared. The concentration of naringenin in media is comparable to that in host pine induced by Chinese-resident fungi [25]. Naringenin was dissolved in ethyl acetate and mixed into molten media to yield the appropriate percentages by dry mass (microgram of naringenin per gram of dry phloem media). Previous study indicated that RTB survivorship was not impacted by ethyl acetate [25].

Tissue samples from 35 galleries were separately washed using 10% PBS buffer to obtain gallery microbiota, which were added to phloem media in 35-mm-diameter Petri dishes and then mixed well. Control phloem media was mixed with 10% PBS buffer. Each Petri dish contained one larva. Beetles were checked daily for mortality. The survival rate of RTB larvae was calculated by Kaplan-Meier survival analysis, and comparisons between survival curves were further tested by Log Rank (Mantel-Cox) method using GraphPad Prism 6 for statistical analyses (GraphPad Prism Software, Inc., San Diego, CA).

### Metagenome sequencing and analysis

In order to sample the gallery microbiome associated with all RTB life stages, 120 samples were extracted from galleries that had recently been in contact with live eggs, young larvae, older larvae, pupae, teneral adults, and attacking adults from host *P. tabuliformis* trees at Tunlanchuan Forestry Station (*n* = 20 for each group). Gallery samples (*n* = 120) were pooled in a 50-ml tube (BD falcon) and buffered in sterile MilliQ water. The tube was sonicated (50/60 Hz, 117 V, 1.0 A; Branson Ultrasonics, Danbury, CT) for 30 s, macerated with a plastic pestle, and vortexed at medium speed for 10 s to separate microbial cells from the gallery. The slurry-like liquid was filtrated through a 100-μm filter (BD falcon) and rinsed with water at least three times, then transferred into another 50-ml tube.

The filtrate was centrifuged at 200×*g* for 5 min at 4 °C. NaCl (at a final concentration of 0.9%) and 10% SDS (at a final concentration of 0.063%) were added to the supernatant followed by incubation at 4 °C for 1 h to obtain automatic precipitation. The upper phase was carefully transferred to a clean bottle while not disturbing the precipitate and filtrated through a 70-μm filter. The filtrate was then centrifuged at 5000×*g* for 10 min (4 °C) to collect the pellet, which was resuspended in 400 ml of MilliQ water. The precipitation step was repeated. The final pellet was suspended in 5 ml of 50 mM Tris-HCl (pH 7.5) and filtrated through a 40-μm filter. Five milliliters of Nycodenz (Nycomed, Oslo, Norway; density approx 1.3 g/ml; 60% *w*/*v* in 50 mM Tris-HCl) was carefully added to 5 ml of the sample (suspension), using a syringe needle of adequate length to reach the bottom of the tube. The sample was then centrifuged in a swing-out rotor (14,000*g* for 40 min at 4 °C). The cell layer was carefully collected. This cell suspension (approximately 500 μl) was mixed with an equal volume of sterile water in a 1.5-ml microtube, centrifuged at 10,000 rpm for 1 min at 4 °C, and used for DNA extraction with Qiagen DNeasy Plant Maxi Kit (Qiagen Sciences, Germantown, MD, USA). DNA sequencing was performed using the Illumina Genome Analyzer at the Beijing Genomics Institute (Shenzhen, China).

The raw sequences obtained from the shotgun library were first quality filtered by trimming adapters, removing low-quality reads, and excluding host contamination. Clean data were then assembled by the SOAP denovo assembler [26], with -d 1 -M 3 -u -F parameters. Different K-mers (49, 55, 59) were tested, and the value was selected based on the largest N50 of the assembled scaffolds. Contigs (continuous sequences within scaffolds) more than 500 bp were retained in the final assembly used for further analyses. For functional annotation, MetaGeneMark [27, 28] was employed to predict the ORFs of the assembled contigs. The predicted ORFs were grouped using CD-HIT with the coverage over 90% and minimum identity 95%, and finally, the nonredundant unigene set was obtained. Unigenes were used to search against the database including all of the microbe genome sequences deposited in GenBank by BLASTn, with *e*-value ≤ minimal *e*-value × 10 for further analysis [29]. The taxonomic level of each unigene was determined by the lowest common ancestor (LCA)-based algorithm that was applied in MEGAN [30]. The taxonomic abundance was evaluated by taking a sum of the number of reads from each taxon, which was obtained by mapping all of the reads to the unigene set using SOAPaligner with default parameters [31]. The unigenes were translated into protein sequences using NCBI Genetic Codes. We used BLASTp to search for the protein sequences of the predicted genes in KEGG, eggNOG, and CAZy databases, with *e*-value ≤ 1e^−5^. The genes with KEGG annotation were assigned into KEGG pathways.

### Characterization of naringenin-biodegrading activity for gallery microbiota

The galleries of beetles from different developmental stages, including young larvae (*n* = 7), older larvae (*n* = 13), pupae (*n* = 11), teneral adult (*n* = 10), and attacking adult stages (*n* = 10), were collected in sterile vials and returned to the lab for experiments. A portion of homogenized gallery sample was immediately stored at − 80 °C in 2-ml cryovials (Corning, USA) for DNA extraction. The other portion was used to evaluate its naringenin-biodegrading activity. Gallery crude extracts were transferred into 1 ml of inorganic culture solution containing 1 mM naringenin in glass test tubes, sealed by silica gel stoppers and incubated for 72 h at 30 °C with shaking (150 rpm). Healthy phloem from six mature *P. tabuliformis* were set as controls and treated the same way as galleries. After 72 h of incubation, remaining quantities of naringenin were detected by high-performance liquid chromatography (HPLC) (Agilent, USA) following previously described procedures [32]. The naringenin-biodegrading activity was expressed as micrograms of naringenin degraded by per gram of (dry weight) gallery tissues.

No significant differences were found in naringenin biodegradation between these stage-grouped galleries, which were all significantly different from healthy phloem (Additional file 1: Figure S1a, *F*_5, 51_ = 4.319, *P* = 0.002). There was obvious individual variation in naringenin-biodegrading activity for each of the gallery groups, from less than 2000 μg g^−1^ DW to near 20,000 μg g^−1^ DW (Additional file 1: Figure S1a). We selected 19 gallery samples across the range of this variation and categorized them by activity level [low (< 2000 μg g^−1^ DW; 6 samples), medium (2000–7000 μg g^− 1^ DW; 6 samples) and high (7000–20,000 μg g^−1^ DW; 7 samples)] (Additional file 1: Figure S1b).

### DNA extraction, PCR, and pyrosequencing

Gallery tissues of the corresponding 19 samples stored at − 80 °C were used to extract genomic DNA separately using E.Z.N.A. Soil DNA Kit (Omega, Bio-Tek, Norcross, GA, USA) following the manufacturer’s instructions. The amount of DNA was determined by Qubit Fluorometer (Invitrogen, USA), and the integrity of DNA was checked by 1% (*w*/*v*) agarose gel electrophoresis stained with ethidium bromide. For analysis of the bacterial community, the hypervariable domains V1–V3 of the 16S rDNA gene were amplified using the primers 27F (5′-AGAGTTTGATCCTGGCTCAG-3′) and 533R (5′-TTACCGCGGCTGCTGGCAC-3′; [33]). The primers 27F and 533R were modified for 454 pyrosequencing with titanium chemistry using the final configuration Adapter A-MID-533R and Adapter B-27F. MIDs were the sample-unique ten bases and established following the rules described [34]. For analysis of the fungal community, the primer set adapted for pyrosequencing consisted of Adapter A-MID-ITS 1 (Internal Transcribed Spacer 1; 5’-TCCGTAGGTGAACCTGCGG-3′) and Adapter B-ITS 4 (5’-TCCTCCGCTTATTGATATGC-3′). For each sample, triplicate PCR reactions were performed and consisted of 0.4 μl of TransStart FastPfu DNA polymerase (TransGen, Biotech, China), 4 μl of 5× FastPfu buffer (TransGen), 2 μl of 2.5 mM dNTPs (TransGen), 0.8 μl of 5 μM of each primer, and 10 ng of template DNA. The ddH_2_O was added to reach the 20 μl reactions.

Template DNA from each sample was used for both bacterial and fungal amplifications. For bacteria, PCR incubations comprised an initial denaturation step at 95 °C for 2 min, followed by 27 cycles of denaturation at 95 °C for 30 s, annealing at 55 °C for 30 s and extension at 72 °C for 30 s and finalized with a 5-min extension step at 72 °C to ensure complete amplification of the target region. For fungi, cycling parameters were 1 cycle of 95 °C for 2 min, 35 cycles of 95 °C for 30 s, 55 °C for 30 s, and 72 °C for 35 s followed by a final extension at 72 °C for 5 min. Negative controls (blank sample and no template) were included in all steps of the process to check for contamination. After pooling amplicons from the triplicate reactions, PCR products were confirmed using 2% agarose gel electrophoresis and cleaned using the AxyPrep DNA Gel Extraction Kit (Axygen Bioscience, CA, USA) following the manufacturer’s instructions. Clean PCR products were quantified using the QuantiFluor dsDNA System kit (Promega) with a QuantiFluor-ST fluorometer. Pyrosequencing of amplicons, using Titanium Lib-L chemistry, was carried out on a 454 Genome Sequencer FLX (Roche) machine installed at BGI (Shenzhen, China).

### Sequence processing

Initial sequence data was processed, assessed for quality, assigned to samples, and analyzed using the QIIME v.1.3.1 software package [35]. Only those reads passing sliding window (50 bp) check (Phred average > 20) with more than 200 bp in length, and no ambiguous characters were included for analyses. Qualified sequences were clustered into operational taxonomic units (OTUs) at 97% sequence similarity using the UPARSE pipeline of USEARCH [36, 37], a similarity level used for both bacteria and fungi [38, 39]. Chimeras were screened using the program UCHIME [40] and removed during the clustering process. The most abundant sequence from each OTU was selected as the representative sequence for that OTU within the cluster. The OTU abundance table was constructed by mapping sequences of all samples to the representative OTUs (-usearch_global-strand plus-id 0.97) and applying the *uc2otutab.py* script. The representative OTUs were assigned taxonomically using the RDP classifier v.2.2 [41] with the SILVA 16S rRNA gene database (v.115) [42] for bacteria and with the UNITE ITS gene database (v.5.0) [43] for fungi. Taxonomical assignments with < 70% confidence threshold were marked as unclassified.

### Pyrosequencing data analyses

Rarefaction curves generated by the *alpha_rarefaction.py* workflow script in QIIME were used to test whether the sequencing efforts adequately represented the bacterial and fungal diversity within each sample. The number of OTUs, Shannon diversity index (H′), Simpson diversity index (1-D), and Buzas and Gibson’s evenness index (e^H′^/S) of each sample was calculated by PAST software [44] and compared across categories (low/medium/high) of naringenin-biodegrading activity. Relative abundances of different phyla and genera in each sample were calculated and compared between activity categories using one-way ANOVA (when data were normally distributed) or Kruskal-Wallis test (when data were not normally distributed).

Jaccard distance matrix for samples of presence-absence OTU data were visualized using NMDS (non-metric multidimensional scaling) plots. Composition differences between activity categories were tested using ANOSIM (analysis of similarity; [45]) with 10,000 permutations. To further investigate the correlations of community composition with naringenin-biodegrading activity, Mantel tests were carried out using Jaccard distances calculated for community data and Euclidean distances for the activity data (continuous data) between samples. The significance of Mantel statistic *r* was given after 1000 permutations. NMDS and ANOSIM were implemented in PAST, and Mantel tests were run in R software with the package *Vegan* [46], based on presence-absence OTU data in communities.

The representative sequences of OTUs were used to construct neighbor-joining trees. The phylogenetic tree together with sample sequence abundance data was used for weighted Unifrac PCoA (principal coordinate analysis) which considers both relative abundance and different branch lengths in a tree, through the online Fast Unifrac program [47]. PERMANOVA tests based on weighted UniFrac distance were run in R software with the package *GUniFrac* [48]. PCo1 data from PCoA plots were tested for correlation with the activity data. Unifrac distances were measured between all pairs of samples. The links between community compositions and naringenin-biodegrading activity (function) were further determined by PLS (partial least square projection to latent structures) with autoscaling in SIMCA-P v.11.5 (Umetrics, Sweden; [49]). *X* variables were all OTUs with relative abundances, and the *Y* variable (response variable) was activity of naringenin degradation. Variables that were suggested to need transformation were log-transformed before analysis.

To identify which OTUs were associated with specific activity groups, we used the INDVAL (indicator value) analysis [50] in R with the package *Labdsv*. Good indicators (OTUs) were those having significant *P* value (*P* < 0.05) and IV (indicator value) > 0.3 [50]. The selected OTUs with strong indicator values were used as key variables to test their predictive ability and fitness for naringenin-biodegrading activity by the PLS model in SIMCA-P. OTUs (or variables) applied to these above analyses were relative abundance data.

### Anti-fungal and anti-bacterial bioassays

Galleries were macerated in 500 μl of 10% PBS buffer and lightly vortexed to obtain crude extracts that contained the microbiota. In the anti-fungal bioassay, crude extracts (100 μl, pipetted from each gallery sample) received one of the three treatments: 100 μl of (1) 10% PBS (as control), (2) nystatin and cycloheximide (NC), (3) streptomycin, penicillin, nystatin, and cycloheximide (SPNC). Twenty-eight RTB galleries were used. In the anti-bacterial bioassay, crude extract (100 μl pipetted) from gallery samples received one of the four treatments: 100 μl of (1) 10% PBS (as control), (2) penicillin (P), (3) streptomycin (S), and (4) streptomycin and penicillin (SP). Seventeen RTB galleries were used. The final concentration of antibiotics was 5 mg/ml. Treatment duration was 12 h at 4 °C. Crude extracts were then transferred to 1 ml of 1 mM naringenin solutions, incubated for 72 h, and then remaining quantities of naringenin were determined by HPLC [25]. Effects of anti-fungal or anti-bacterial treatments on the abundances of bacteria and fungi were tested. After treatments, diluted crude extracts were spread on LB and PDA (potato dextrose agar). Single colonies for each possible species of microbe (identified initially by colony morphology) were recorded, selected, and then streaked for three times. Pure strains were identified using previously described methods [25]. One-way ANOVA was carried out for these randomized block designs with gallery sample and treatment as two main effects, followed by S-N-K (Student-Newman-Keul’s) test for multiple comparisons.

Effects of anti-fungal and anti-bacterial treatments on naringenin degradation were compared using both liquid and solid media. Crude extract (100 μl pipetted) from gallery samples received one of the two treatments: 100 μl of (1) nystatin and cycloheximide (NC) and (2) streptomycin and penicillin (SP). Liquid conditions were 1 ml of 1 mM naringenin solutions as described above. Solid conditions were 50 μl of 1 mM naringenin solutions dried on the surfaces of PDA media with crude extracts spread on it. Six RTB galleries were used. The parametric paired-sample *T* test was used to compare the quantity of remaining naringenin following exposure to the crude extracts between the two treatments.

### Beetle survivorship bioassay with *Novosphingobium* sp.

Phloem media amended with naringenin (2000 μg g^− 1^ of media dry weight) was prepared. *Novosphingobium* sp. is a strong naringenin-biodegrading bacterium that has been isolated from RTB galleries [25]. Here, this bacterium was cultured in LB and the cells were washed with 10% PBS buffer before adding to phloem media in 35-mm-diameter Petri dishes. The control phloem media were mixed with 10% PBS buffer. Each Petri dish contained one larva. Twenty RTB larvae were applied for each treatment and beetles were checked daily for mortality. Comparisons between the survival curves were tested by Log Rank (Mantel-Cox) method as described before.

## Results

### Impact of symbiotic microbiota in gallery on beetle survivorship under naringenin

**Fig. 1:**
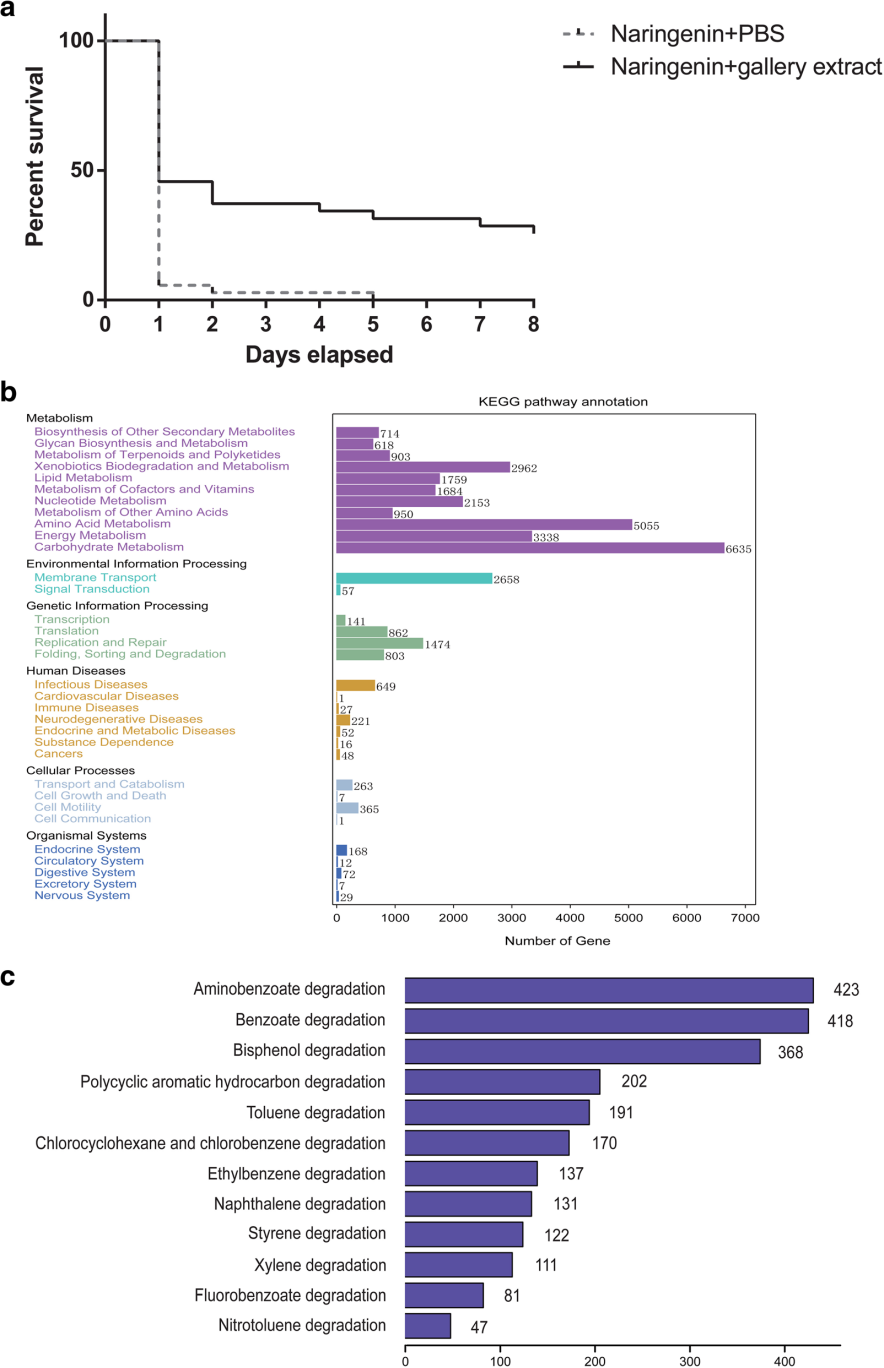
The symbiotic naringenin degrading microbiota in the gallery protect the invasive RTB from naringenin. **a** Survival curves for RTB larvae under naringenin, associated with or without gallery microbiota. **b** KEGG pathway annotation for gallery microbiota. **c** KEGG pathways of aromatics degradation in the xenobiotics biodegradation and metabolism category

In the presence of naringenin (2000 μg g^−1^ of media), the number of live RTB larvae was reduced during the observing time. However, the survival rate for beetles in media without gallery microbiota was significantly lower than those in media with gallery microbiota (Fig. 1a; Mantel-Cox test, *χ*^2^_1_ = 17.73, *P* < 0.0001).

### Metagenomic analysis for the symbiotic microbiota in RTB gallery

We obtained 105,366,604 reads (8.83 Gb in total) from genomic DNA of microbiota in RTB galleries for analysis, after filtering out low-quality reads, adapters, and host contamination. These high-quality reads were assembled into 43,723 contigs (≥ 500 bp), totaling 48.7 Mb. The average length of all contigs was 1167.61 bp, while contig N50 length was 1304 bp, and max scaffold length was 50,431 bp. A total of 75,271 open reading frames (ORFs) were predicted from assembled contigs, including 35.92% complete and 64.08% partial ORFs. After cluster analysis by CD-HIT, we obtained 47,241 non-redundant unigenes. We annotated protein function encoded by these unigenes through BLAST searches. In total, 34,704 unigenes (73.5%) were assigned to 4352 KEGG orthologs (KOs). Among them, metabolisms of carbohydrate, amino acid, energy, and xenobiotics biodegradation were the four top abundant categories (Fig. 1b).

A total of 2962 unigenes (8.5%) were involved in xenobiotics biodegradation and metabolism. In this category, 1291 unigenes (43.6%) were annotated to degrade a variety of aromatic compounds, including aminobenzoate, benzoate, and bisphenol (Fig. 1c). Benzoate degradation and its related pathways are intermediate pathways for degradation of phenolic compounds, which could be further degraded into fatty acids and the terminal product acetyl-CoA could be utilized in the citrate cycle metabolic pathway. Extradiol dioxygenases involved in aromatic ring cleavage and hydroxylation are important enzymes for degrading aromatics into fatty acids. The coding genes for these enzymes were found in the metagenomics of beetle gallery microbiota, assigned to genera such as *Burkholderia*, *Sphingomonas*, *Mycobacterium*, and *Novosphingobium* (Additional file 1: Table S1).

### The relationship between microbial abundances and naringenin degradation

For bacteria in the 19 gallery samples with differential naringenin degrading activities, we obtained a total of 155,786 sequences (91.2% of the total trimmed 170,817 sequences) that could be clustered into 708 OTUs (mean = 186 per sample, s.d. = 52, range = 94–288) at a 97% similarity level. For fungi, 277,369 sequences (96.0% of the total trimmed 288,932 sequences) were obtained among these samples that could be clustered into 209 OTUs (mean = 66 per sample, s.d. = 16, range = 42–100) at a 97% similarity level. The diversities of both bacteria and fungi within each sample were fully characterized, as the rarefaction curves had already reached stable values under the sequencing depth for each sample (Additional file 1: Figure S2). No significant differences were found in number of OTUs, Shannon diversity index, Simpson’s diversity index, and Buzas or Gibson’s evenness index among the three levels (low/medium/high) of naringenin degrading activity, for either bacterial communities (Additional file 1: Table S2) or fungal communities (Additional file 1: Table S3).

**Fig. 2:**
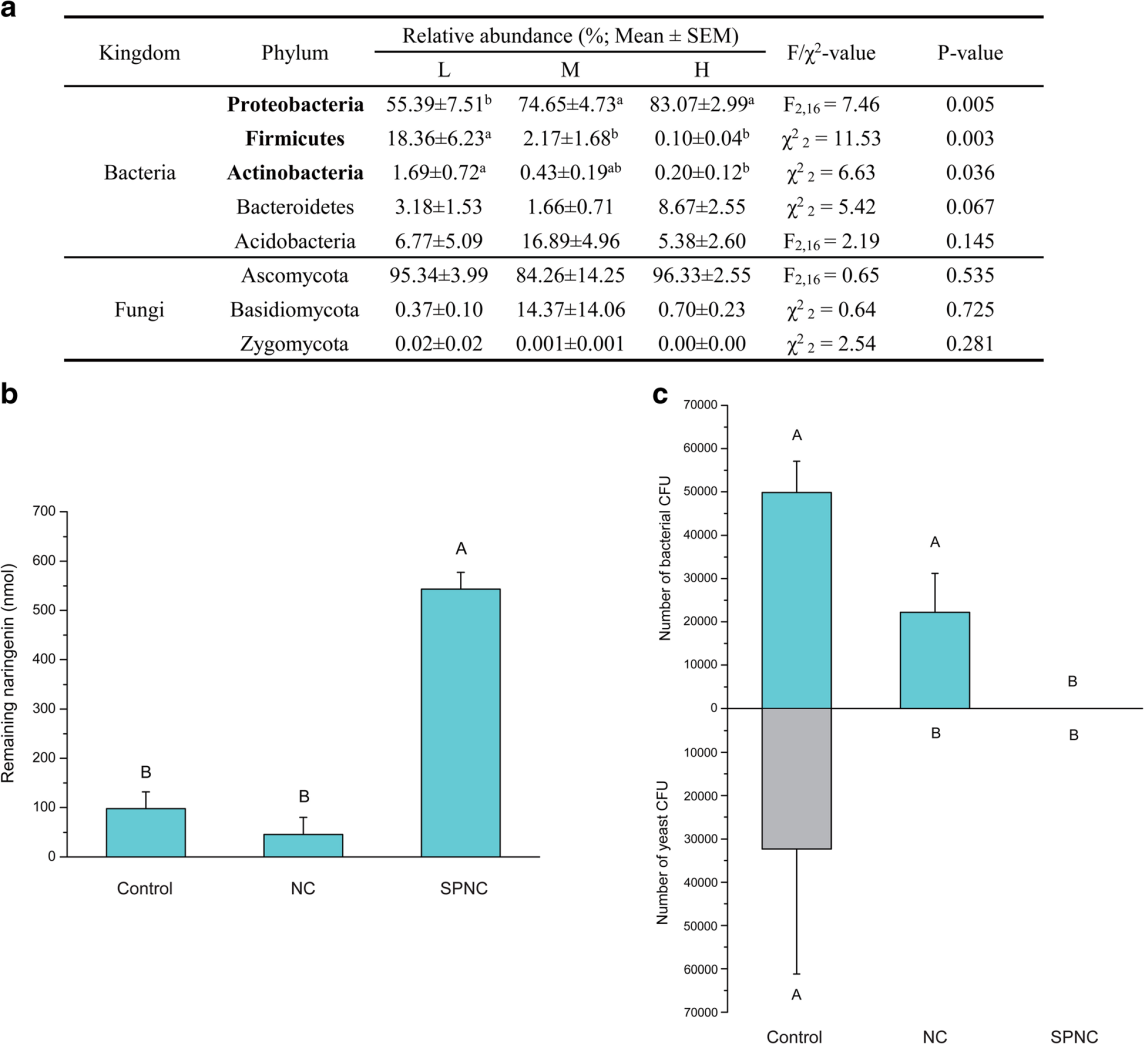
Fungi of RTB gallery microbiota are little involved in naringenin degradation. **a** Phylum-level comparison between low (L), medium (M), and high (H) naringenin degrading activity groups of the symbiotic microbiota in RTB gallery. Low taxonomical level comparisons between the three activity groups were presented in Table S4 and S5 in Additional file 1. Effects of anti-fungal treatment on naringenin degradation (**b**) and bacterial/yeast abundances (**c**). Control: no antibiotic treatment; NC: nystatin and cycloheximide; SPNC: streptomycin, penicillin, nystatin, and cycloheximide

Three fungal phyla that were the most abundant in the fungal community, including Ascomycota, Basidiomycota, and Zygomycota, were not significantly different between activity groups (Fig. 2a). No significant differences between activity groups were found for fungi at the class or order level (Additional file 1: Table S4). However, shifts in bacterial abundances at the phylum level were significant among activity groups. The relative abundances of Proteobacteria were increased significantly in the high naringenin degrading activity group (*P* = 0.005; Fig. 2a), in contrast to significant declines in relative abundances of Firmicutes and Actinobacteria (*P* = 0.003 and *P* = 0.036, respectively; Fig. 2a). Relative abundances of the other two phyla, Bacteroidetes and Acidobacteria, were not found to be statistically different among the three activity groups (*P* = 0.067 and *P* = 0.145, respectively; Fig. 2a). Sixty-seven genera were identified, accounting for an average of 76.3% of all OTUs sequences (Additional file 1: Table S5). Five genera (*Novosphingobium* and *Stenotrophomonas* in particular, *P* = 0.001 and 0.003, respectively), belonging to Proteobacteria, were significantly more abundant in the high activity group (Additional file 1: Table S5). Three genera (especially *Lactococcus* and *Leuconostoc*, *P* = 0.002 and 0.015, respectively) in Firmicutes and *Microbacterium* (*P* = 0.035) in Actinobacteria were significantly more abundant in the low activity group (Additional file 1: Table S5).

### Effects of anti-fungal agents on naringenin degradation by gallery microbiota

No significant difference in naringenin degrading activity was found between the anti-fungal treatment and the control with no antibiotics, but treatment with both anti-bacterial and anti-fungal agents caused significantly higher quantity of remaining naringenin than other treatments (Fig. 2b; *F*_2, 54_ = 64.065, *P* < 0.0001). Treatment with anti-bacterial agents alone resulted in a significantly higher quantity of naringenin remaining compared to the treatment with anti-fungal agents (Additional file 1: Figure S3).

For fungi, only yeasts were isolated from gallery extracts with these treatments. Anti-fungal treatment significantly reduced the number of yeast colony-forming unit (CFU) (Fig. 2c; *F*_2, 4_ = 60.750, *P* = 0.001), but did not have significant effects on the number of bacterial CFUs (Fig. 2c; *F*_2, 4_ = 20.857, *P* = 0.008).

### The relationship between bacterial community composition and naringenin degradation

**Fig. 3:**
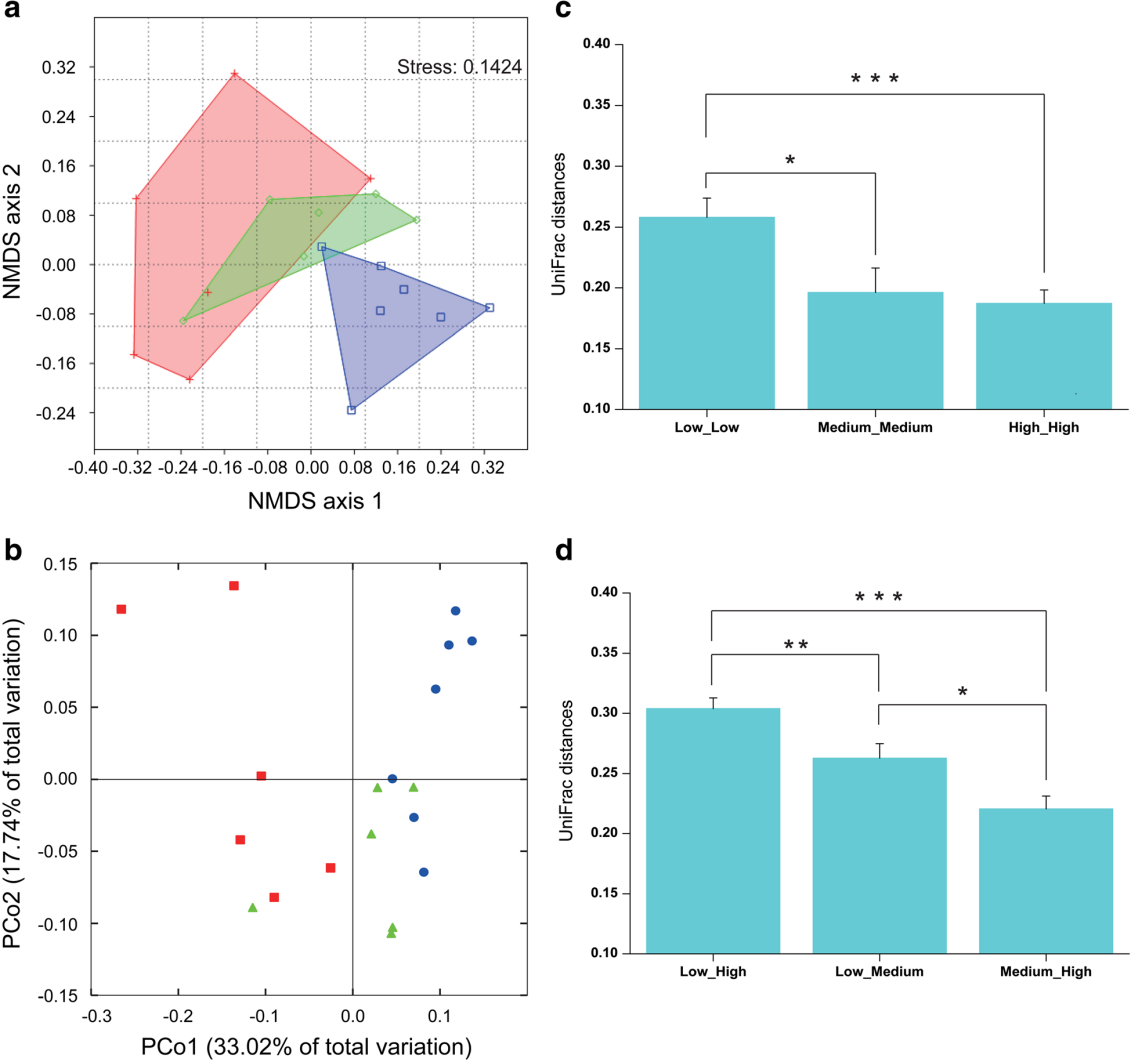
Bacterial community composition correlates to naringenin degradation. **a** Non-metric multidimensional scaling (NMDS) diagrams of the 19 gallery samples based on Jaccard distance matrix, for bacterial communities consisting of OTUs (97% similarity level) as presence-absence data. Samples in low activity group are denoted as red cross, in medium activity group as green diamond and in high activity group as blue square. **b** Principal coordinate analysis (PCoA) plots based on the weighted UniFrac metric for bacterial communities. Red squares, green triangles, and blue circles represent low, medium, and high activity groups, respectively. **c** Weighted UniFrac pairwise distances between samples within each of the three activity groups. **d** Weighted UniFrac pairwise distances between activity groups. Pairwise comparisons were conducted by independent-samples *T* test. * *P* < 0.05, ** *P* < 0.01, *** *P* < 0.001

The large number of sequences were represented by only a few bacterial OTUs, as shown on the ranked relative abundance curves (only 22 bacterial OTUs found in > 1% relative abundance; Additional file 1: Figure S4). Presence-absence OTU data were initially used to describe the bacterial community, which equally emphasized the roles of rare and abundant members in shaping community structure. Bacterial communities were significantly separated among the three activity groups of naringenin biodegradation, which was evident in the NMDS diagram calculated with Jaccard distance (Fig. 3a; ANOSIM, *P* = 0.0016). Samples from the high activity group possessed distinct bacterial OTU composition compared to those from medium and low activity groups, while there was no difference in bacterial community composition between medium and low activity samples (Additional file 1: Table S6). Mantel tests further corroborated a significant correlation between bacterial community composition and naringenin degrading activity (Pearson *r* = 0.26, *P* = 0.02).

The phylogeny-based weighted UniFrac principal coordinate analyses considering relative abundances of OTUs gave similar results (Fig. 3b; PERMANOVA, *P* < 0.001; Regression between PCo1 and naringenin degrading activity, *P* < 0.0001). The weighted UniFrac metric from bacterial communities was used to examine pair-wise distances between the samples according to their origins and groups. Large inter-sample variation was found in the phylogenetic configuration of bacterial communities in the low activity group compared to those in either the medium or high activity group (Fig. 3c), suggesting close similarity of phylotype combinations in the high activity group. The greatest pair-wise distance was between low activity and high activity groups, and the distance was lowest between medium and high activity groups (Fig. 3d).

PLS models were further used to determine how much variation of naringenin degrading activity (*Y* variable) was explained by the relative abundance of bacterial OTUs (*X* variables). The PLS model using bacterial OTUs as *X* variables exhibited high goodness of fit (*R*^2^ = 0.996) and goodness of prediction (*Q*^2^ = 0.796) (Additional file 1: Table S7).

### The relationship between phylotypes in bacterial community and naringenin degradation

**Fig. 4:**
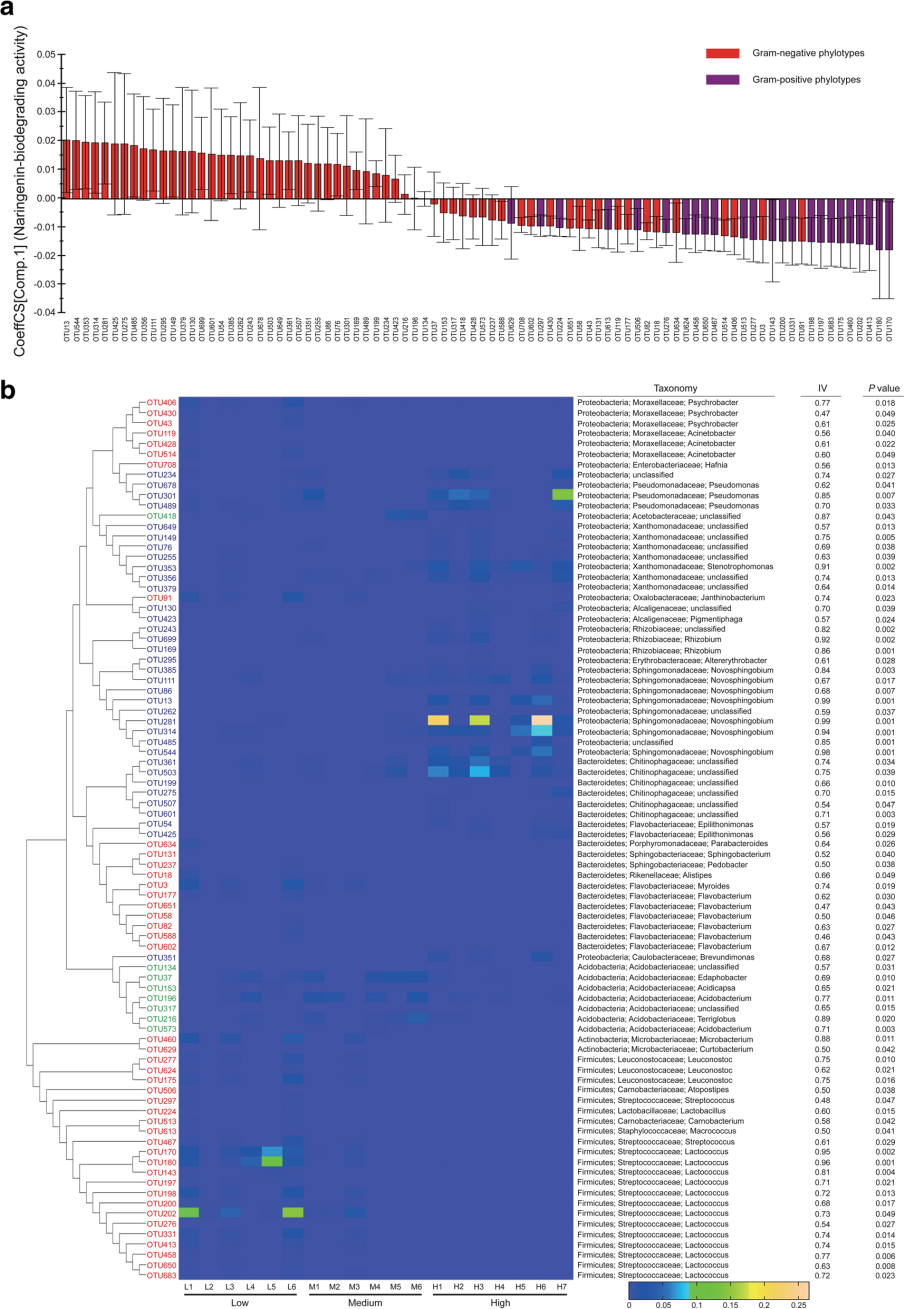
Bacterial indicator phylotypes correlate to naringenin degradation. **a** Plot of regression coefficients of 86 bacterial OTUs identified by INDVAL in PLS model using naringenin-biodegrading activities as the response variable. Columns with positive values mean that the OTUs are positively correlated to the response variable and vice versa. Columns in red mean Gram-negative OTUs and columns in purple mean Gram-positive. Error bars with 95% confidence intervals that do not cross the horizontal axis mean statistically significant coefficients. **b** Relative abundances of the 86 OTUs as indicator phylotypes that characterize specific activity groups. OTUs shown in red color are those specialized in the low activity group, shown in green are the ones characterizing medium activity group and shown in blue are those more specifically enriched in high activity group. Neighbor-joining phylogenetic tree of the OTUs is shown on the left and corresponding taxonomy information (by RDP classifier) is shown on the right. IV: indicator value. Samples are arranged along their activity levels as denoted at the bottom. The color intensity of each OTU means its relative abundance in each sample

Using INDVAL, we detected 86 bacterial OTUs that seemed to be strong indicator phylotypes (both IV > 0.3 and *P* < 0.05), which represented 12.1% of the total bacterial OTU dataset (Fig. 4; Additional file 1: Table S8). The PLS model confirmed that the 86 bacterial OTUs were key *X* variables that explained 93.7% of the variation in naringenin degrading activity (*R*^2^), with a predictive ability (*Q*^2^) of 58.8% (Additional file 1: Table S7).

Among these indicator phylotypes, 43 and 35 OTUs were specifically distributed in the low and high activity groups, respectively; 8 OTUs significantly characterized the medium activity group (Additional file 1: Table S8). In the low activity group, top indicators included three OTUs (IV 0.96, 0.95, and 0.81) from *Lactococcus* (Firmicutes) and one OTU (IV 0.88) from *Microbacterium* (Actinobacteria) (Fig. 4b). In the medium activity group, majority of the indicators were affiliated with the family Acidobacteriaceae (Fig. 4b). In the high activity group, all indicators were Gram-negative and were positively correlated with naringenin degrading activity (Fig. 4a). The overall Gram-negative bacteria taxa were positively correlated with naringenin biodegradation (Additional file 1: Figure S5), which possessed abundant genes in the category of xenobiotics biodegradation (Additional file 1: Figure S6).

A large amount of indicators in the high activity group (27 out of 35) were mapped to phylum Proteobacteria with the top ones belonging to the genus *Novosphingobium* (Fig. 4b); one *Novosphingobium* OTU (IV 0.99) had an average relative abundance of over 10% in total high activity communities and even accounted for around 20% of the sequences in some samples (Fig. 4b; Additional file 1: Table S8).

### Impact of *Novosphingobium* species on beetle survivorship under naringenin

**Fig. 5:**
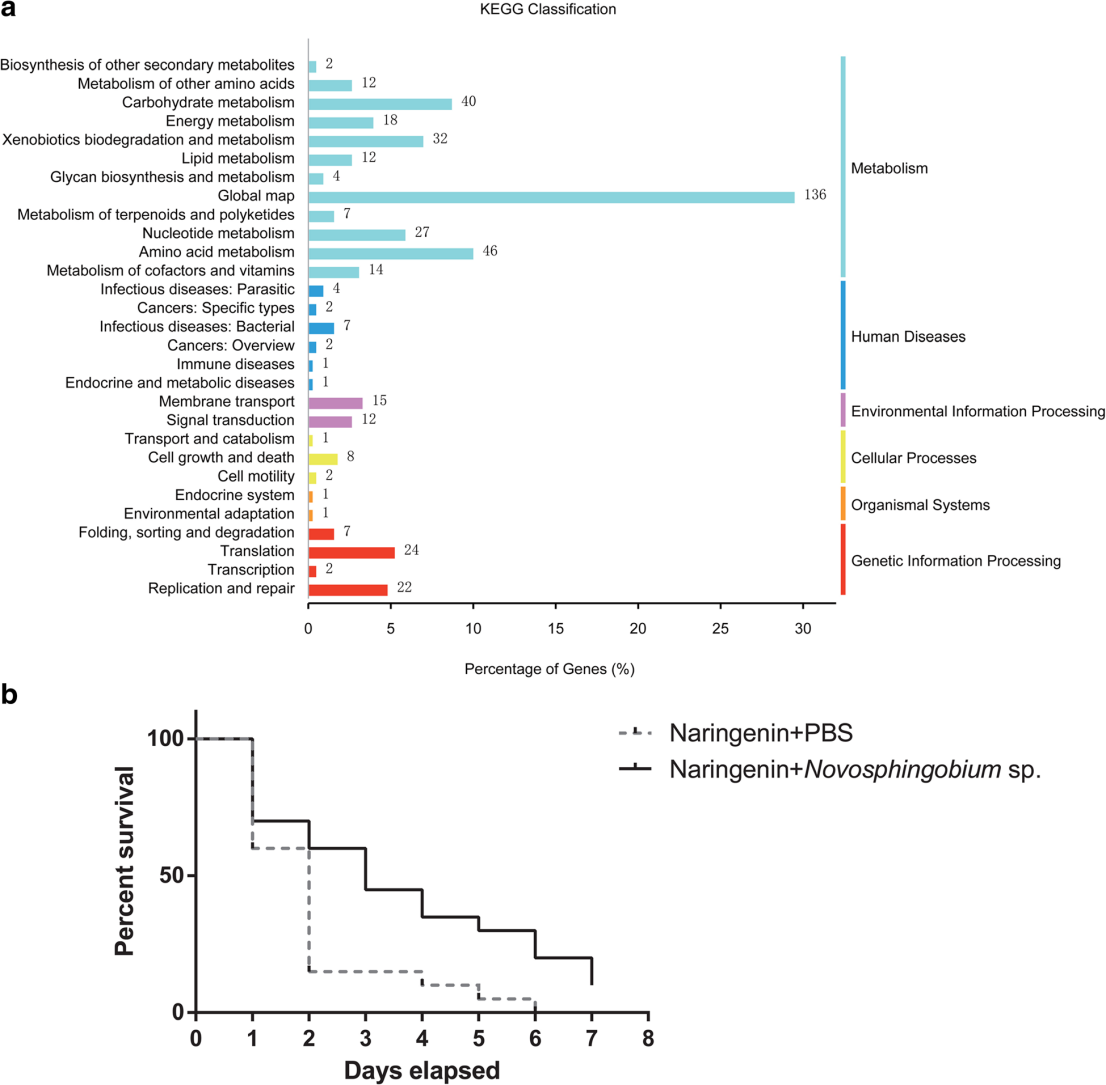
Association with bacterial species in *Novosphingobium* help protect the invasive RTB from naringenin. **a** KEGG pathway annotation for *Novosphingobium* enriched in Gram-negatives. **b** Survival curves for RTB larvae under naringenin, associated with or without *Novosphingobium* sp.

For *Novosphingobium*, 276 unigenes were assigned to 108 KEGG orthologs (KOs). Among them, global map, amino acid metabolism, carbohydrate metabolism, and xenobiotics biodegradation and metabolism were the top four abundant categories (Fig. 5a). The global map category contained three pathways, including metabolic pathways (77 genes), microbial metabolism in diverse environments (33 genes), and biosynthesis of secondary metabolites (26 genes). *Novosphingobium* possessed putative genes involved in metabolism of diverse aromatic compounds, the metabolic products of which were suggested to be further utilized by other metabolic pathways (Table 1; Additional files 2 and 3).

**Table 1:**
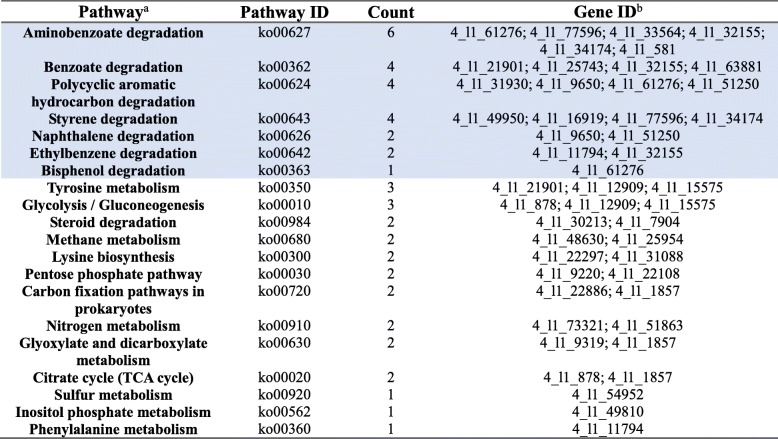
Genes involved in metabolism of diverse aromatic compounds and other connected metabolisms by *Novosphingobium*

^a^Pathway interactions were presented in Additional file 2; Pathways for aromatics degradations were included in the blue highlighting;

^b^Gene IDs for microbial metabolism in diverse environments was provided by a .html file in Additional file 3

Furthermore, for beetles living in media supplemented with naringenin (2000 μg g^−1^ of media), the survival rate for beetles without *Novosphingobium* sp. was significantly lower than those in media associated with this bacterium (Fig. 5b; Mantel-Cox test, *χ*^2^_1_ = 6.03, *P* = 0.014).

Our study presented here, based on metagenomics and 454 pyrosequencing data together with the corresponding beetle bioassay results, seeks to identify key microbial players of symbiotic microbiota in the RTB gallery that protect the invasive beetle from naringenin, an inducible defensive compound of the host pine. First, through the beetle-survivorship bioassay, symbiotic naringenin-degrading microbiota were found to increase RTB survivorship (Fig. 5b), and potential genes in degradation pathways were discovered by metagenomics (Fig. 1). Second, the anti-fungal bioassay demonstrated that fungi had little involvement in naringenin degradation (Fig. 2), while bacterial community structure was highly correlated to naringenin degrading activity (Fig. 3). Last, phylotypes of genus *Novosphingobium*, Gram-negative bacteria, were extraordinarily correlated to the degrading activity, which were determined to possess putative genes involved in degradation pathways by metagenomics analysis (Figs. 4 and 5). The beetle-survivorship bioassay confirmed that RTB associated with an isolated species of this genus acquired protection against naringenin (Fig. 5).

## Discussion

The naringenin degradation function was highly correlated with bacterial community structure in the microbiota. It is not surprising that fungi in the symbiotic microbiota of the RTB galleries had little involvement in naringenin degradation (Fig. 2). The majority of fungal species belonged to yeasts (around 80% of fungal reads were affiliated to order Saccharomycetales; Additional file 1: Table S4). The most two highly abundant OTUs in the fungal communities together made up 46.4% of reads (Additional file 1: Figure S4b) and these two fungal OTUs matched to the yeast species *Cyberlindnera americana* CBS5644 at similarity of 97.2 and 100%, respectively. Yeast species have been isolated from RTB galleries of different developmental stages in the field [25, 51]. Yeasts exhibiting strong naringenin degrading activities were infrequently associated with RTB, and the most frequently isolated yeasts (e.g., *C. americana*) had the lowest potential to degrade naringenin among the yeasts isolated from RTB galleries [25]. Metagenomic data also revealed that putative genes functioning in aromatic ring cleavage were assigned to bacteria rather than yeasts (Additional file 1: Table S1). Fungi may play a minor role in naringenin degradation as indicated by the combined anti-fungal/anti-bacterial treatment having the least naringenin degradation. However, our data demonstrated that the bacterial community alone, evidenced by presence of only bacteria colony forming units in the anti-fungal treatment, had a stronger correlation with the degradation function (Fig. 2b, c).

The taxonomy- and OTU-level compositions of bacterial communities were significantly divergent among activity groups (Figs. 2a and 3). Phyla Firmicutes and Actinobacteria were more abundant in the low activity group compared to the high activity group of galleries; conversely, phylum Proteobacteria was more abundant in the high activity group than in the low activity group of galleries (Fig. 2a). Abundances of 86 bacterial phylotypes explained most of the variation in naringenin degradation by symbiotic microbiota (Additional file 1: Table S7). Phylotypes belonging to the Gram-negatives were positively correlated to naringenin degrading activity (Fig. 4a), and Gram-negative phylotypes were mainly enriched in the high activity group of RTB galleries (Fig. 4b). Metagenomic analysis revealed abundant putative genes for biodegradation in Gram-negatives (Additional file 1: Figure S6). Thus, we hypothesized that phylotypes in Gram-negative bacteria of galleries protect RTB from naringenin.

*Novosphingobium* is a genus of Gram-negative bacteria, and the phylotypes found in this genus are expected to degrade naringenin. A bacterial isolate from sediment of Lake Tai, identified to be a novel member of the genus *Novosphingobium*, assimilated several aromatic compounds such as phenol, aniline, nitrobenzene, 4-chloronitrobenzene, and phenanthrene [52]. Isolates of this genus from marine sediments were also shown to be capable of degrading multiple polycyclic aromatic hydrocarbons (PAHs) [53, 54]. The presence of *Novosphingobium* sp. HS2aR improved the growth of plants in phenanthrene-contaminated soil by degradation [55]. In our system, through metagenomic analysis, *Novosphingobium* was found to possess putative genes involved in metabolic pathways for multiple aromatic compounds degradation which were linked to down-stream carbohydrate, lipid, and amino acid metabolisms (Fig. 5a; Table 1). One gene encoding aromatic ring hydroxylating dioxygenase, which is important for aromatic ring cleavages [56], was identified from *Novosphingobium* (Additional file 1: Table S1). *Novosphingobium* sp. has been isolated from RTB gallery, one of the gallery microbes exhibiting strong naringenin degrading activity [25]. Here, in the beetle-survivorship bioassay, RTB larvae associated with this bacterium acquired protection against naringenin (Fig. 5b).

The symbiotic microbiota exhibited different naringenin degrading activities in RTB galleries (Additional file 1: Figure S1). This variation of naringenin degrading activity in gallery communities may be attributed to differences of the abundance of key bacteria like *Novosphingobium*, or by interactions with other microbiota. In our work, OTUs 385, 111, 86, 13, 281, 314, and 544 were assigned to the genus *Novosphingobium* and their abundances/frequencies in different naringenin-biodegrading groups are shown in Fig. 4 and Additional file 1: Table S8.

It is of interest to know why gallery communities varied and why certain naringenin-degrading bacterial microbes, such as *Novosphingobium* sp., became dominant in the high activity group, while had low abundance in low activity group. We suspect two factors may have resulted in this variation: First, nearly half of the low-activity indicator groups belonged to the order Lactobacillales, which can produce lactic acid as the major metabolic end-product of carbohydrate fermentation, suggesting a possible low pH condition in low activity galleries. On the contrary, these acid-producing taxa were nearly absent in high activity galleries, which implies a distinctive pH condition in the gallery microenvironment may facilitate growth of *Novosphingobium* bacteria. Second, the change in host pine defensive chemistry may also influence the abundances of microorganisms residing in galleries. In the case of host pines infected with Chinese-resident fungi during the course of RTB attack, pine phloem can be stimulated to produce high amounts of naringenin. Many bacterial taxa cannot tolerate high levels of naringenin, while this condition is very suitable for bacteria in the genus *Novosphingobium*, which are capable of degrading this compound and use as its sole carbon source. Nevertheless, it may be the invasion process of RTB itself that facilitates the favorable gallery microbiome conditions resulting in the preponderance of *Novosphingobium* in the high activity group. Interactions between microbes within a community and physiochemical conditions of microenvironments as important ecological factors [57, 58], may ultimately shape the differential gallery communities that lead to varied degrading activities. These potential factors will be elucidated in future studies.

Microbial symbionts that are essential for the invasion success of weeds and pests usually share common traits: they are frequently associated with hosts, predominant in niches, and exhibit functions that enhance their hosts’ invasiveness; and importantly, inflict a severe fitness cost to individuals lacking the symbiont. In fact, the ultimate functions of microbes, as extended phenotypes for exotic invaders, are likely context-dependent and may change with environmental conditions and the presence of other cooperating microbes in the community assemblage. Given these reasons, key players of symbiotic microbiota involved in facilitating the success of species invasion should be elucidated from the community level down to the particular taxa. Our results presented here could enlighten researchers on providing complementary insights into assessing “essential symbionts” that are involved in dynamic symbiotic associations with invasive species.

## Conclusions

In the present study, symbiotic naringenin-degrading microbiota in the gallery were found to protect invasive RTB from naringenin, a phenolic defensive chemical in host pines. The naringenin degradation function was highly correlated with bacterial community structure in the microbiota. This study might be among the first attempts of combining a variety of bioassay results with metagenomics and 454 pyrosequencing data to demonstrate the roles of key symbiotic microbes in facilitating species invasion. In this process, the overall symbiotic microbiota were used for beetle survivorship bioassay, the naringenin-degrading function was evidenced by metagenomics, and then the functions of bacteria and fungi in the microbiota were indicated by the anti-fungal treatment bioassays. The function of bacterial microbiota was further confirmed by 454 pyrosequencing data analyses and metagenomics, and finally, a representative bacterial strain was used in a beetle survivorship bioassay to validate the protective role of microbiota against naringenin. This research elucidated the key players of symbiotic microbiota from the community level down to the particular taxa, involved in facilitating RTB invasions, and exemplifies a good case study to further understand other successful symbiotic invasion systems.

## Additional files

Additional file 1: This file includes: **Table S1.** eggNOG function annotation for dioxygenases in aromatics degradation. **Table S2.** Comparison of diversity indices (Mean ± SEM) between bacterial gallery microbiota of low (L), medium (M), and high (H) naringenin biodegrading activity. **Table S3.** Comparison of diversity indices (Mean ± SEM) between fungal gallery microbiota of low (L), medium (M), and high (H) naringenin biodegrading activity. **Table S4.** Class- and order-level comparisons between fungal communities in RTB galleries of low (L), medium (M), and high (H) naringenin degrading activity. **Table S5.** Changes in relative abundances of main bacterial genera with reported known functions in biodegradation. **Table S6.** ANOSIM *R* values between naringenin biodegrading activity groups. **Table S7.** Model statistics of PLS. **Table S8.** Detailed information for the 86 indicator phylotypes. **Figure S1.** Naringenin-biodegrading activity (Mean ± SEM) of RTB galleries with large variation between samples. **Figure S2.** Rarefaction curves of the 19 samples for bacterial OTUs and fungal OTUs. **Figure S3.** Effects of anti-fungal and anti-bacterial treatments on naringenin degradation, under liquid and solid media condition. **Figure S4.** The rank abundance diagram of the 708 bacterial OTUs and 209 fungal OTUs identified, plotted as the traditional Whittaker plot. **Figure S5.** Effects of anti-bacterial treatments on naringenin degradation and abundance of Gram-negative bacteria. **Figure S6.** KEGG pathway annotations for Gram-negative bacteria and Gram-positive bacteria in RTB gallery microbiota. (DOC 5448 kb)

Additional file 2: map01120**.** Projection of genes involved in microbial metabolism in diverse environments on the KEGG pathways. (PNG 276 kb)

Additional file 3: Gene IDs of KEGG pathways in map01120. (HTML 848 kb)

## Abbreviations

ANOSIM: Analysis of similarity
CFU: Colony-forming unit
HPLC: High-performance liquid chromatography
INDVAL: Indicator value
KO: KEGG ortholog
LCA: Lowest common ancestor
NMDS: Non-metric multidimensional scaling
ORF: Open reading frames
OTU: Operational taxonomic unit
PBS: Phosphate buffered saline
PCoA: Principal coordinate analysis
PDA: Potato dextrose agar
PLS: Partial least square projection to latent structures
RTB: Red turpentine beetle
S-N-K: Student-Newman-Keul
