# Supplementary material for: Bacterial microbiota protect an invasive bark beetle from a pine defensive compound
Source: Microbiome. 2018 Jul 27;6:132. doi: 10.1186/s40168-018-0518-0 (PMC6064089; doi:10.1186/s40168-018-0518-0)
Supplement: Supplementary file 1 — This file includes: Table S1. eggNOG function annotation for dioxygenases in aromatics degradation. Table S2. Comparison of diversity indices (Mean ± SEM) between bacterial gallery microbiota of low (L), medium (M), and high (H) naringenin biodegrading activity. Table S3. Comparison of diversity indices (Mean ± SEM) between fungal gallery microbiota of low (L), medium (M), and high (H) naringenin biodegrading activity. Table S4. Class- and order-level comparisons between fungal communities in RTB galleries of low (L), medium (M), and high (H) naringenin degrading activity. Table S5. Changes in relative abundances of main bacterial genera with reported known functions in biodegradation. Table S6. ANOSIM R values between naringenin biodegrading activity groups. Table S7. Model statistics of PLS. Table S8. Detailed information for the 86 indicator phylotypes. Figure S1. Naringenin-biodegrading activity (Mean ± SEM) of RTB galleries with large variation between samples. Figure S2. Rarefaction curves of the 19 samples for bacterial OTUs and fungal OTUs. Figure S3. Effects of anti-fungal and anti-bacterial treatments on naringenin degradation, under liquid and solid media condition. Figure S4. The rank abundance diagram of the 708 bacterial OTUs and 209 fungal OTUs identified, plotted as the traditional Whittaker plot. Figure S5. Effects of anti-bacterial treatments on naringenin degradation and abundance of Gram-negative bacteria. Figure S6. KEGG pathway annotations for Gram-negative bacteria and Gram-positive bacteria in RTB gallery microbiota. (DOC 5448 kb) [file 40168_2018_518_MOESM1_ESM.doc]

**Additional file 1**

**Bacterial microbiota protect an invasive bark beetle from a pine defensive compound**

Chihang Cheng, Jacob Wickham, Li Chen, Dandan Xu, Min Lu, Jianghua Sun

Corresponding author: Min Lu and Jianghua Sun

Email: lumin@ioz.ac.cn; sunjh@ioz.ac.cn

Additional file 1 includes: Table S1, Table S2, Table S3, Table S4, Table S5, Table S6, Table S7, Table S8, Figure S1, Figure S2, Figure S3, Figure S4, Figure S5, Figure S6.

| **Orthologous Group** | **Function description** | **Gene numbers** | | **Taxa affiliation** |
| --- | --- | --- | --- | --- |
| **COG3485** | Protocatechuate 3,4-dioxygenase | 17 | *Burkholderia* (1), *Olivibacter* (1), *Sphingomonas* (1), *Terriglobus* (3), unknown (11) | |
| **COG3384** | Extradiol ring-cleavage dioxygenase class IIi protein subunit b | 15 | *Caulobacter* (1), *Chryseobacterium* (1), *Sphingomonas* (1), unknown (12) | |
| **COG3435** | Gentisate 1,2-dioxygenase | 5 | *Mycobacterium* (1), *Sphingomonas* (1), unknown (3) | |
| **COG3508** | Homogentisate 1,2-dioxygenase | 5 | Unknown (5) | |
| **ENOG4111UDS** | Aromatic-ring-hydroxylating dioxygenase beta subunit | 5 | *Sphingopyxis* (1), unknown (4) | |
| **ENOG410YY4Z** | Extradiol ring-cleavage dioxygenase LigAB, LigA subunit | 1 | Unknown (1) | |
| **COG3885** | Extradiol ring-cleavage dioxygenase class IIi protein subunit b | 1 | *Pusillimonas* (1) | |
| **ENOG4111ISH** | Catechol 2,3-dioxygenase | 1 | Unknown (1) | |
| **ENOG410XS2Y** | Extradiol ring-cleavage dioxygenase | 1 | Unknown (1) | |
| **ENOG410YX8U** | Aromatic-ring-hydroxylating dioxygenase subunit beta | 1 | *Novosphingobium* (1) | |

**Table S1** eggNOG function annotation for dioxygenases in aromatics degradation.

**Table S2** Comparison of diversity indices (Mean ± SEM) between bacterial gallery microbiota of low (L), medium (M), and high (H) naringenin biodegrading activity.

| Index | L | M | H | F_2,16_ | *P*-value |
| --- | --- | --- | --- | --- | --- |
| Number of OTUs | 217.17±25.61 | 186.17±20.36 | 158.14±11.67 | 2.38 | 0.124 |
| Shannon diversity (H’) | 3.22±0.23 | 3.12±0.19 | 3.35±0.19 | 0.35 | 0.711 |
| Simpson’s diversity (1-D) | 0.90±0.03 | 0.88±0.03 | 0.91±0.03 | 0.36 | 0.704 |
| Buzas and Gibson's evenness  (e^H’^/S) | 0.12±0.01 | 0.14±0.02 | 0.19±0.02 | 3.49 | 0.055 |

**Table S3** Comparison of diversity indices (Mean ± SEM) between fungal gallery microbiota of low (L), medium (M), and high (H) naringenin biodegrading activity.

| Index | L | M | H | F_2,16_ | *P*-value |
| --- | --- | --- | --- | --- | --- |
| Number of OTUs | 64.67±7.83 | 63.00±7.36 | 70.00±4.65 | 0.32 | 0.729 |
| Shannon diversity (H’) | 1.91±0.19 | 1.80±0.17 | 2.08±0.18 | 0.68 | 0.519 |
| Simpson’s diversity (1-D) | 0.76±0.03 | 0.71±0.05 | 0.78±0.03 | 0.94 | 0.410 |
| Buzas and Gibson's evenness  (e^H’^/S) | 0.11±0.01 | 0.11±0.02 | 0.12±0.02 | 0.29 | 0.753 |

**Table S4** Class- and order-level comparisons between fungal communities in RTB galleries of low (L), medium (M), and high (H) naringenin degrading activity.

| Class | Relative abundance (%; Mean ± SEM) | | | F/χ^2^-value | P-value |
| --- | --- | --- | --- | --- | --- |
|  | L | M | H |  |  |
| Agaricomycetes | 0.14±0.10 | 13.90±13.90 | 0.05±0.05 | χ^2^ _2_ = 3.315 | 0.191 |
| Agaricostilbomycetes | 0.00±0.00 | 0.00±0.00 | 0.02±0.02 | χ^2^ _2_ = 2.069 | 0.355 |
| Ascomycota_norank | 0.00±0.00 | 0.02±0.01 | 0.00±0.00 | F_2,16_ = 3.408 | 0.059 |
| Dothideomycetes | 0.33±0.14 | 0.60±0.48 | 0.12±0.11 | F_2,16_ = 0.727 | 0.499 |
| Eurotiomycetes | 0.00±0.00 | 0.11±0.11 | 0.00±0.00 | χ^2^ _2_ = 2.318 | 0.314 |
| Fungi_norank | 0.02±0.01 | 0.10±0.08 | 0.02±0.02 | χ^2^ _2_ = 1.584 | 0.453 |
| Incertae | 0.02±0.02 | 0.00±0.00 | 0.00±0.00 | χ^2^ _2_ = 2.540 | 0.281 |
| Leotiomycetes | 0.02±0.01 | 15.63±15.30 | 6.51±6.00 | χ^2^ _2_ = 2.903 | 0.234 |
| Microbotryomycetes | 0.21±0.10 | 0.46±0.17 | 0.62±0.23 | F_2,16_ = 1.298 | 0.300 |
| Saccharomycetes | 92.81±3.99 | 59.84±16.80 | 88.47±6.00 | χ^2^ _2_ = 3.156 | 0.206 |
| Sordariomycetes | 0.01±0.01 | 0.02±0.02 | 0.08±0.04 | χ^2^ _2_ = 2.850 | 0.241 |
| Tremellomycetes | 0.02±0.02 | 0.00±0.00 | 0.01±0.01 | χ^2^ _2_ = 2.372 | 0.305 |
| Unclassified | 6.42±3.93 | 9.31±5.61 | 4.10±2.52 | F_2,16_ = 0.420 | 0.664 |

| Order | Relative abundance (%; Mean ± SEM) | | | F/χ^2^-value | P-value |
| --- | --- | --- | --- | --- | --- |
|  | L | M | H |  |  |
| Agaricales | 0.02±0.01 | 0.00±0.00 | 0.00±0.00 | χ^2^ _2_ = 4.574 | 0.102 |
| Agaricostilbales | 0.00±0.00 | 0.00±0.00 | 0.02±0.02 | χ^2^ _2_ = 2.069 | 0.355 |
| Ascomycota_norank | 0.00±0.00 | 0.02±0.01 | 0.00±0.00 | F_2,16_ = 3.408 | 0.059 |
| Boletales | 0.02±0.02 | 0.00±0.00 | 0.00±0.00 | χ^2^ _2_ = 2.167 | 0.338 |
| Cantharellales | 0.10±0.10 | 13.90±13.90 | 0.05±0.05 | χ^2^ _2_ = 0.077 | 0.962 |
| Capnodiales | 0.30±0.12 | 0.60±0.48 | 0.12±0.11 | F_2,16_ = 0.750 | 0.488 |
| Chaetothyriales | 0.00±0.00 | 0.11±0.11 | 0.00±0.00 | χ^2^ _2_ = 2.318 | 0.314 |
| Cystofilobasidiales | 0.01±0.01 | 0.00±0.00 | 0.00±0.00 | χ^2^ _2_ = 2.167 | 0.338 |
| Fungi_norank | 0.02±0.01 | 0.10±0.08 | 0.02±0.02 | χ^2^ _2_ = 1.584 | 0.453 |
| Helotiales | 0.02±0.01 | 15.63±15.30 | 6.51±6.00 | χ^2^ _2_ = 2.903 | 0.234 |
| Hypocreales | 0.00±0.00 | 0.00±0.00 | 0.08±0.04 | F_2,16_ = 3.389 | 0.059 |
| Incertae | 0.00±0.00 | 0.00±0.00 | 0.003±0.003 | χ^2^ _2_ = 1.714 | 0.424 |
| Leucosporidiales | 0.003±0.003 | 0.00±0.00 | 0.00±0.00 | χ^2^ _2_ = 2.167 | 0.338 |
| Ophiostomatales | 0.01±0.01 | 0.02±0.02 | 0.00±0.00 | χ^2^ _2_ = 2.540 | 0.281 |
| Pleosporales | 0.03±0.03 | 0.00±0.00 | 0.00±0.00 | χ^2^ _2_ = 2.167 | 0.338 |
| Saccharomycetales | 92.81±3.99 | 59.84±16.80 | 88.47±6.00 | χ^2^ _2_ = 3.156 | 0.206 |
| Sporidiobolales | 0.20±0.11 | 0.32±0.08 | 0.61±0.23 | F_2,16_ = 1.771 | 0.202 |
| Tremellales | 0.01±0.01 | 0.00±0.00 | 0.01±0.01 | F_2,16_ = 0.513 | 0.608 |
| Unclassified | 6.44±3.92 | 9.46±5.57 | 4.10±2.52 | F_2,16_ = 0.447 | 0.647 |

**Table S5** Changes in relative abundances of main bacterial genera with reported known functions in biodegradation.

| Genus^a^ | Relative abundance (%; Mean **±** SEM) | | | F/χ^2^-value | P-value | Reference | Known activity to degrade aromatic compounds |
| --- | --- | --- | --- | --- | --- | --- | --- |
|  | L | M | H |  |  |  |  |
| *Frateuria* | 12.66±8.56 | 26.02±11.09 | 14.69±8.12 | F_2,16_ = 0.58 | 0.569 | 1 | aniline |
| *Rhodanobacter* | 4.33±3.81 | 4.86±1.94 | 1.52±1.03 | F_2,16_ = 0.58 | 0.574 | 2 | benzopyrene |
| *Pseudoxanthomonas* | 1.38±1.28 | 2.48±0.86 | 6.59±2.27 | χ^2^ _2_ = 4.38 | 0.112 | 3 | benzene, toluene, ethylbenzene, and xylene |
| ***Stenotrophomonas*** | 0.10±0.03 | 0.15±0.09 | 2.28±0.52 | χ^2^ _2_ = 11.46 | 0.003 | 4 | *p*-nitrophenol and 4-chlorophenol |
| *Luteibacter* | 0.007±0.004 | - | 0.17±0.16 | χ^2^ _2_ = 4.97 | 0.083 | 5 | polychlorinated biphenyl |
| *Dyella* | 0.78±0.71 | 0.21±0.19 | 0.05±0.05 | χ^2^ _2_ = 2.83 | 0.242 | 6 | biphenyl |
| *Hydrocarboniphaga* | 0.002±0.002 | 0.007±0.005 | 0.21±0.13 | χ^2^ _2_ = 3.59 | 0.167 | 7 | aromatic hydrocarbons |
| *Pseudomonas* | 5.67±3.24 | 1.24±0.73 | 8.43±3.70 | χ^2^ _2_ = 3.38 | 0.185 | 8 | gallic acid (phenolics) |
| *Acinetobacter* | 0.40±0.23 | 0.05±0.04 | 0.006±0.004 | χ^2^ _2_ = 5.48 | 0.065 | 9 | phenol |
| ***Psychrobacter*** | 0.69±0.39 | 0.06±0.05 | 0.003±0.002 | χ^2^ _2_ = 6.47 | 0.039 | 10 | low-ethoxylated nonylphenols |
| *Enhydrobacter* | 0.64±0.38 | 0.06±0.04 | 0.001±0.001 | χ^2^ _2_ = 4.99 | 0.083 | - | **-** |
| *Nevskia* | 0.28±0.22 | 6.10±4.07 | 0.07±0.04 | χ^2^ _2_ = 1.26 | 0.534 | - | **-** |
| *Rahnella* | 5.12±4.43 | 4.62±2.72 | 4.21±1.77 | F_2,16_ = 0.02 | 0.977 | - | **-** |
| *Serratia* | 0.13±0.07 | 0.04±0.03 | 0.05±0.03 | F_2,16_ = 1.13 | 0.347 | 11 | tannic acid |
| *Yersinia* | 0.12±0.08 | 0.03±0.02 | 0.04±0.02 | F_2,16_ = 0.91 | 0.423 | - | **-** |
| *Pantoea* | 0.02±0.01 | 0.18±0.17 | 0.004±0.004 | χ^2^ _2_ = 1.86 | 0.395 | 11 | tannic acid |
| *Erwinia* | 4.29±4.28 | 0.007±0.005 | 0.001±0.001 | χ^2^ _2_ = 2.35 | 0.310 | 12 | aniline |
| *Burkholderia* | 1.65±1.01 | 5.54±1.77 | 4.09±1.15 | F_2,16_ = 2.06 | 0.160 | 13 | chlorophenols |
| *Pandoraea* | 0.005±0.003 | 0.002±0.002 | 0.27±0.26 | χ^2^ _2_ = 0.64 | 0.727 | 14 | polychlorinated biphenyl |
| *Ralstonia* | 0.04±0.02 | 0.01±0.01 | - | χ^2^ _2_ = 5.04 | 0.080 | 15 | 2,4,6-trichlorophenol |
| *Variovorax* | 0.003±0.003 | 0.01±0.01 | 0.08±0.07 | χ^2^ _2_ = 1.54 | 0.464 | 16 | homovanillate (phenolics) |
| *Comamonas* | 0.05±0.03 | 0.01±0.01 | 1.50±1.46 | χ^2^ _2_ = 1.30 | 0.523 | 17 | 4-chlorophenol and phenol |
| *Delftia* | 0.003±0.003 | - | 0.03±0.03 | χ^2^ _2_ = 1.97 | 0.374 | 18 | phenolic compounds |
| *Candidimonas* | 0.02±0.02 | 0.25±0.23 | 0.47±0.24 | χ^2^ _2_ = 5.84 | 0.054 | - | **-** |
| *Achromobacter* | 0.01±0.01 | 0.002±0.002 | 0.10±0.09 | χ^2^ _2_ = 2.03 | 0.362 | 19 | *p*-nitrophenol |
| ***Pigmentiphaga*** | - | - | 0.12±0.08 | χ^2^ _2_ = 8.09 | 0.018 | 20 | 2,6-Naphthalenedisulfonate |
| ***Janthinobacterium*** | 0.49±0.26 | 0.07±0.05 | - | χ^2^ _2_ = 9.48 | 0.009 | 21 | 2,4-dinitrophenol |
| *Herbaspirillum* | - | 0.01±0.01 | 0.06±0.04 | χ^2^ _2_ = 2.07 | 0.355 | 22 | fluoranthene |
| ***Novosphingobium*** | 0.21±0.14 | 0.41±0.21 | 17.72±6.66 | χ^2^ _2_ = 13.14 | 0.001 | 23 | polychlorophenols |
| *Sphingomonas* | 0.29±0.15 | 1.18±0.67 | 1.44±0.55 | χ^2^ _2_ = 4.38 | 0.112 | 24 | pentachlorophenol |
| *Sphingobium* | 0.002±0.002 | 0.01±0.01 | 0.20±0.13 | χ^2^ _2_ = 3.00 | 0.223 | 25 | pentachlorophenol |
| ***Altererythrobacter*** | 0.005±0.003 | 0.01±0.01 | 0.04±0.01 | χ^2^ _2_ = 6.33 | 0.042 | 26 | aromatic compounds |
| *Acetobacter* | 1.67±1.20 | 0.43±0.13 | 0.76±0.74 | F_2,16_ = 0.60 | 0.562 | - | **-** |
| *Acidisoma* | 1.46±0.89 | 2.21±1.57 | 0.86±0.23 | F_2,16_ = 0.47 | 0.632 | - | **-** |
| *Acidocella* | 0.47±0.42 | 6.65±3.84 | 0.39±0.23 | χ^2^ _2_ = 2.24 | 0.326 | 27 | salicylic acid (phenolics) |
| *Gluconobacter* | 0.33±0.26 | 0.05±0.01 | 0.04±0.03 | χ^2^ _2_ = 4.53 | 0.104 | - | **-** |
| *Bradyrhizobium* | 0.05±0.03 | 0.03±0.01 | 0.05±0.02 | F_2,16_ = 0.38 | 0.688 | 28 | catechin (phenolics) |
| ***Rhizobium*** | 0.002±0.002 | 0.03±0.02 | 0.45±0.20 | χ^2^ _2_ = 12.57 | 0.002 | 29 | 4-chlorophenol |
| *Methylovirgula* | 0.06±0.05 | 0.33±0.19 | 0.06±0.04 | χ^2^ _2_ = 1.11 | 0.574 | - | **-** |
| *Methylobacterium* | 0.01±0.01 | 0.003±0.003 | 0.01±0.01 | F_2,16_ = 0.37 | 0.695 | 30 | dimethyl isophthalate |
| *Ochrobactrum* | 0.02±0.02 | 0.002±0.002 | 0.06±0.05 | F_2,16_ = 0.63 | 0.543 | 14 | polychlorinated biphenyl |
| *Brevundimonas* | 0.03±0.01 | 0.003±0.003 | 0.54±0.29 | χ^2^ _2_ = 5.93 | 0.052 | 31 | isoquinoline (phenolics) |
| *Acidobacterium* | 5.19±4.07 | 11.22±3.10 | 4.01±2.23 | F_2,16_ = 1.51 | 0.252 | 32 | benzene |
| *Granulicella* | 0.35±0.24 | 0.92±0.50 | 0.73±0.28 | χ^2^ _2_ = 1.11 | 0.573 | - | **-** |
| *Edaphobacter* | 0.37±0.18 | 1.15±0.36 | 0.14±0.07 | χ^2^ _2_ = 3.78 | 0.151 | - | **-** |
| *Terriglobus* | 0.15±0.11 | 1.48±1.05 | 0.10±0.03 | χ^2^ _2_ = 3.37 | 0.185 | - | **-** |
| ***Acidicapsa*** | 0.03±0.02 | 0.16±0.06 | 0.004±0.003 | χ^2^ _2_ = 6.97 | 0.031 | - | **-** |
| *Nocardioides* | 0.02±0.01 | 0.02±0.01 | 0.09±0.05 | χ^2^ _2_ = 0.58 | 0.747 | 33 | *p*-nitrophenol and phenol |
| ***Microbacterium*** | 1.17±0.65 | 0.16±0.14 | 0.04±0.03 | χ^2^ _2_ = 6.72 | 0.035 | - | **-** |
| *Acidothermus* | 0.06±0.06 | 0.02±0.02 | - | χ^2^ _2_ = 1.24 | 0.538 | - | **-** |
| *Mycobacterium* | 0.04±0.02 | 0.10±0.08 | 0.003±0.003 | χ^2^ _2_ = 3.60 | 0.165 | 34 | chlorinated phenolic compounds |
| *Pseudonocardia* | 0.05±0.03 | 0.01±0.01 | 0.004±0.004 | χ^2^ _2_ = 1.07 | 0.585 | 35 | monofluorophenols |
| ***Lactococcus*** | 15.79±5.03 | 1.69±1.29 | 0.05±0.03 | χ^2^ _2_ = 12.75 | 0.002 | - | **-** |
| *Streptococcus* | 0.61±0.34 | 0.06±0.05 | 0.007±0.004 | χ^2^ _2_ = 3.28 | 0.194 | - | **-** |
| ***Leuconostoc*** | 1.07±0.58 | 0.12±0.08 | 0.001±0.001 | χ^2^ _2_ = 8.45 | 0.015 | - | **-** |
| ***Enterococcus*** | 0.12±0.05 | 0.04±0.01 | - | χ^2^ _2_ = 9.06 | 0.011 | - | **-** |
| *Brochothrix* | 0.45±0.25 | 0.06±0.05 | 0.01±0.01 | χ^2^ _2_ = 3.87 | 0.144 | - | **-** |
| ***Bacteroides*** | 0.77±0.64 | 0.49±0.48 | - | χ^2^ _2_ = 7.27 | 0.026 | - | **-** |
| ***Alistipes*** | 0.25±0.16 | 0.003±0.002 | - | χ^2^ _2_ = 6.76 | 0.034 | - | **-** |
| ***Parabacteroides*** | 0.17±0.13 | 0.02±0.02 | - | χ^2^ _2_ = 6.51 | 0.039 | - | **-** |
| *Chryseobacterium* | 0.20±0.10 | 0.03±0.03 | 0.59±0.36 | χ^2^ _2_ = 2.71 | 0.258 | - | **-** |
| *Flavobacterium* | 0.46±0.26 | 0.05±0.04 | 0.43±0.42 | F_2,16_ = 0.53 | 0.600 | 36 | chlorinated phenols |
| *Epilithonimonas* | 0.01±0.01 | 0.002±0.002 | 0.46±0.19 | χ^2^ _2_ = 3.94 | 0.139 | - | **-** |
| ***Myroides*** | 0.93±0.52 | 0.12±0.10 | 0.001±0.001 | χ^2^ _2_ = 7.77 | 0.021 | 37 | 3,4-dichloroaniline |
| *Mucilaginibacter* | 0.04±0.02 | 0.20±0.13 | 0.31±0.16 | χ^2^ _2_ = 1.93 | 0.382 | - | **-** |
| *Sphingobacterium* | 0.05±0.02 | 0.01±0.01 | 0.75±0.66 | χ^2^ _2_ = 1.24 | 0.538 | 38 | lignin monomer (phenolics) |
| *Pedobacter* | 0.03±0.02 | 0.003±0.003 | 0.30±0.29 | χ^2^ _2_ = 1.92 | 0.382 | - | **-** |

^a^ Genera with bold names are the ones with significant differences in relative abundance between activity groups.

**Table S6** ANOSIM *R* values^a^ between naringenin biodegrading activity groups.

| **Bacterial community** | | |
| --- | --- | --- |
|  | **Medium** | **High** |
| **Low** | 0.1037(NS) | 0.6045** |
| **Medium** |  | 0.2844* |

^a^ *R* values were calculated using 10000 permutations based on Jaccard distance matrix. *R* values ~ 0 indicate no separation between groups and *R* values > 0.5 indicate good separation. Significance for *R* value is Bonferroni corrected: ** *p* < 0.01, * *p* < 0.05; NS, No Significance.

**Table S7** Model statistics of PLS^a,b^.

| Explanatory variables (X) | Response variable (Y) | *R^2^*^c^ | *Q^2^*^d^ | NO. of OTUs | N^e^ |
| --- | --- | --- | --- | --- | --- |
| Bacterial OTUs | Naringenin biodegrading activity | 0.996 | 0.796 | 708 | 4 |
| Identified key bacterial OTUs |  | 0.937 | 0.588 | 86 | 3 |

^a^ Each model is based on the relative abundances of OTUs that describe the response variable measured for each gallery sample;

^b^ PLS, partial least square projection to latent structures;

^c^ *R^2^*, goodness of fit;

^d^ *Q^2^*, goodness of prediction;

^e^ N, number of PLS components.

**Table S8 Detailed information for the 86 indicator phylotypes.**

| Significant indicator | Represented activity group | Indicator value (IV) | *P* value | Relative abundance (and % frequency) in each group | | |
| --- | --- | --- | --- | --- | --- | --- |
|  |  |  |  | Low | Medium | High |
| OTU3 | Low | 0.74 | 0.019 | 0.0092 (83%) | 0.0012 (50%) | 2.0E-05 (14%) |
| OTU18 | Low | 0.66 | 0.049 | 0.002 (67%) | 3.4E-05 (33%) | 0 |
| OTU43 | Low | 0.61 | 0.025 | 0.0014 (67%) | 0.0001 (17%) | 2.0E-05 (14%) |
| OTU58 | Low | 0.50 | 0.046 | 5.9E-05 (50%) | 0 | 0 |
| OTU82 | Low | 0.63 | 0.027 | 0.0005 (67%) | 3.1E-05 (17%) | 0 |
| OTU91 | Low | 0.74 | 0.023 | 0.0049 (83%) | 0.0007 (67%) | 0 |
| OTU119 | Low | 0.56 | 0.040 | 0.0006 (67%) | 0.0001 (17%) | 0 |
| OTU131 | Low | 0.52 | 0.040 | 0.0003 (67%) | 9.3E-05 (17%) | 0 |
| OTU143 | Low | 0.81 | 0.004 | 0.0011 (83%) | 3.5E-05 (33%) | 0 |
| OTU170 | Low | 0.95 | 0.002 | 0.0261 (100%) | 0.0013 (83%) | 6.0E-05 (29%) |
| OTU175 | Low | 0.75 | 0.016 | 0.0048 (83%) | 0.0006 (50%) | 2.0E-05 (14%) |
| OTU177 | Low | 0.62 | 0.030 | 0.0021 (67%) | 0.0001 (17%) | 6.0E-05 (14%) |
| OTU180 | Low | 0.96 | 0.001 | 0.0346 (100%) | 0.0012 (83%) | 6.3E-05 (29%) |
| OTU197 | Low | 0.71 | 0.021 | 0.0014 (83%) | 0.0003 (67%) | 2.0E-05 (14%) |
| OTU198 | Low | 0.72 | 0.013 | 0.0078 (83%) | 0.0013 (50%) | 0 |
| OTU200 | Low | 0.68 | 0.017 | 0.0014 (83%) | 0.0004 (50%) | 0 |
| OTU202 | Low | 0.73 | 0.049 | 0.0483 (83%) | 0.007 (83%) | 9.9E-05 (29%) |
| OTU224 | Low | 0.60 | 0.015 | 0.0005 (67%) | 6.2E-05 (17%) | 0 |
| OTU237 | Low | 0.50 | 0.038 | 0.0003 (50%) | 0 | 0 |
| OTU276 | Low | 0.54 | 0.027 | 0.0003 (67%) | 8.2E-05 (33%) | 0 |
| OTU277 | Low | 0.75 | 0.010 | 0.0032 (83%) | 0.0004 (33%) | 0 |
| OTU297 | Low | 0.48 | 0.047 | 0.0009 (50%) | 3.1E-05 (17%) | 0 |
| OTU331 | Low | 0.74 | 0.014 | 0.0053 (83%) | 0.0007 (50%) | 0 |
| OTU406 | Low | 0.77 | 0.018 | 0.0042 (83%) | 0.0004 (50%) | 2.0E-05 (14%) |
| OTU413 | Low | 0.74 | 0.015 | 0.0031 (83%) | 0.0004 (67%) | 0 |
| OTU428 | Low | 0.61 | 0.022 | 0.0003 (67%) | 0 | 4.8E-05 (14%) |
| OTU430 | Low | 0.47 | 0.049 | 0.0013 (50%) | 9.3E-05 (17%) | 0 |
| OTU458 | Low | 0.77 | 0.006 | 0.0007 (83%) | 4.7E-05 (17%) | 2.0E-05 (14%) |
| OTU460 | Low | 0.88 | 0.011 | 0.0111 (100%) | 0.0016 (83%) | 5.9E-05 (14%) |
| OTU467 | Low | 0.61 | 0.029 | 0.0035 (67%) | 0.0003 (33%) | 0 |
| OTU506 | Low | 0.50 | 0.038 | 4.8E-05 (50%) | 0 | 0 |
| OTU513 | Low | 0.58 | 0.042 | 0.0008 (67%) | 0.0001 (50%) | 0 |
| OTU514 | Low | 0.60 | 0.049 | 0.0016 (67%) | 0.0002 (50%) | 0 |
| OTU588 | Low | 0.46 | 0.043 | 0.0009 (50%) | 9.3E-05 (17%) | 0 |
| OTU602 | Low | 0.67 | 0.012 | 0.0002 (67%) | 0 | 0 |
| OTU613 | Low | 0.50 | 0.041 | 0.0001 (50%) | 0 | 0 |
| OTU624 | Low | 0.62 | 0.021 | 0.0008 (67%) | 6.2E-05 (17%) | 0 |
| OTU629 | Low | 0.50 | 0.042 | 0.0001 (50%) | 0 | 0 |
| OTU634 | Low | 0.64 | 0.026 | 0.0014 (67%) | 6.2E-05 (17%) | 0 |
| OTU650 | Low | 0.63 | 0.008 | 0.0009 (67%) | 6.2E-05 (17%) | 0 |
| OTU651 | Low | 0.47 | 0.043 | 0.0003 (50%) | 2.2E-05 (17%) | 0 |
| OTU683 | Low | 0.72 | 0.023 | 0.0018 (83%) | 0.0003 (50%) | 0 |
| OTU708 | Low | 0.56 | 0.013 | 0.0002 (67%) | 1.6E-05 (17%) | 4.0E-05 (14%) |
| OTU37 | Medium | 0.69 | 0.010 | 0.0034 (67%) | 0.0104 (100%) | 0.001 (86%) |
| OTU134 | Medium | 0.57 | 0.031 | 2.8E-05 (17%) | 0.0002 (67%) | 2.4E-05 (14%) |
| OTU153 | Medium | 0.65 | 0.021 | 0.0003 (50%) | 0.0014 (83%) | 4.5E-05 (29%) |
| OTU196 | Medium | 0.77 | 0.011 | 0.0026 (67%) | 0.0106 (100%) | 0.0014 (86%) |
| OTU216 | Medium | 0.89 | 0.020 | 0.0008 (50%) | 0.0099 (100%) | 0.0007 (100%) |
| OTU317 | Medium | 0.65 | 0.015 | 0.0001 (33%) | 0.0006 (83%) | 4.5E-05 (14%) |
| OTU418 | Medium | 0.87 | 0.043 | 0.0008 (83%) | 0.0079 (100%) | 0.0004 (43%) |
| OTU573 | Medium | 0.71 | 0.003 | 0.0002 (17%) | 0.0007 (83%) | 0 |
| OTU13 | High | 0.99 | 0.001 | 1.50E-05 (17%) | 0.0001 (33%) | 0.0161 (100%) |
| OTU54 | High | 0.57 | 0.019 | 0 | 0 | 0.0019 (57%) |
| OTU76 | High | 0.69 | 0.038 | 1.50E-05 (17%) | 0.0004 (67%) | 0.0019 (86%) |
| OTU86 | High | 0.68 | 0.007 | 0 | 2.10E-05 (17%) | 0.0005 (71%) |
| OTU111 | High | 0.67 | 0.017 | 0.001 (33%) | 0.0022 (100%) | 0.0069 (100%) |
| OTU130 | High | 0.70 | 0.039 | 5.80E-05 (33%) | 0.0003 (67%) | 0.0019 (86%) |
| OTU149 | High | 0.75 | 0.005 | 7.50E-05 (17%) | 0.0004 (67%) | 0.002 (100%) |
| OTU169 | High | 0.86 | 0.001 | 0 | 0 | 0.0004 (86%) |
| OTU199 | High | 0.66 | 0.010 | 0 | 2.20E-05 (17%) | 0.0003 (71%) |
| OTU234 | High | 0.74 | 0.027 | 0.0009 (83%) | 0.0008 (67%) | 0.0075 (100%) |
| OTU243 | High | 0.82 | 0.002 | 1.50E-05 (17%) | 3.60E-05 (33%) | 0.0019 (86%) |
| OTU255 | High | 0.63 | 0.039 | 0.0003 (33%) | 0.0004 (67%) | 0.0029 (86%) |
| OTU262 | High | 0.59 | 0.037 | 0.0003 (50%) | 0.0002 (33%) | 0.0015 (86%) |
| OTU275 | High | 0.70 | 0.015 | 0 | 3.80E-05 (33%) | 0.0024 (71%) |
| OTU281 | High | 0.99 | 0.001 | 0.0004 (67%) | 0.0006 (83%) | 0.1014 (100%) |
| OTU295 | High | 0.61 | 0.028 | 4.30E-05 (33%) | 0.0001 (33%) | 0.0004 (86%) |
| OTU301 | High | 0.85 | 0.007 | 0.0011 (67%) | 0.004 (67%) | 0.0393 (100%) |
| OTU314 | High | 0.94 | 0.001 | 0.0006 (67%) | 0.001 (50%) | 0.0288 (100%) |
| OTU351 | High | 0.68 | 0.027 | 0.0001 (50%) | 0 | 0.0042 (71%) |
| OTU353 | High | 0.91 | 0.002 | 0.0002 (33%) | 0.0013 (67%) | 0.0184 (100%) |
| OTU356 | High | 0.74 | 0.013 | 0.0005 (33%) | 0.0015 (100%) | 0.0078 (100%) |
| OTU361 | High | 0.74 | 0.034 | 0.0006 (50%) | 0.0018 (83%) | 0.0164 (86%) |
| OTU379 | High | 0.64 | 0.014 | 0 | 0.0002 (33%) | 0.0006 (86%) |
| OTU385 | High | 0.84 | 0.003 | 0 | 4.60E-05 (17%) | 0.003 (86%) |
| OTU423 | High | 0.57 | 0.024 | 0 | 0 | 0.0012 (57%) |
| OTU425 | High | 0.56 | 0.029 | 0 | 2.30E-05 (17%) | 0.0024 (57%) |
| OTU485 | High | 0.85 | 0.001 | 0 | 2.30E-05 (17%) | 0.0033 (86%) |
| OTU489 | High | 0.70 | 0.033 | 0.0016 (50%) | 0.0013 (67%) | 0.0111 (100%) |
| OTU503 | High | 0.75 | 0.039 | 0.0008 (67%) | 0.0027 (83%) | 0.0285 (86%) |
| OTU507 | High | 0.54 | 0.047 | 1.50E-05 (17%) | 0 | 0.0004 (57%) |
| OTU544 | High | 0.98 | 0.001 | 8.60E-05 (50%) | 0.0002 (83%) | 0.0203 (100%) |
| OTU601 | High | 0.71 | 0.003 | 0 | 0 | 0.0005 (71%) |
| OTU649 | High | 0.57 | 0.013 | 0 | 0 | 0.0004 (57%) |
| OTU678 | High | 0.62 | 0.041 | 0.0001 (50%) | 3.70E-05 (33%) | 0.0008 (86%) |
| OTU699 | High | 0.92 | 0.002 | 1.50E-05 (17%) | 0.0003 (50%) | 0.0041 (100%) |

**Figure S1.** Naringenin-biodegrading activity (Mean ± SEM) of RTB galleries with large variation between samples. (**a**) Heterogeneous biodegrading activities of galleries from each RTB developmental stage. Different letters on error bars mean significant difference between groups. Numbers in parentheses mean sample size for each group. (**b**) Nineteen representative gallery samples selected from those in (a) with significant gradient in naringenin biodegradation. Samples are categorized into three groups according to biodegrading activities: low (6 samples), medium (6 samples) and high (7 samples).

**
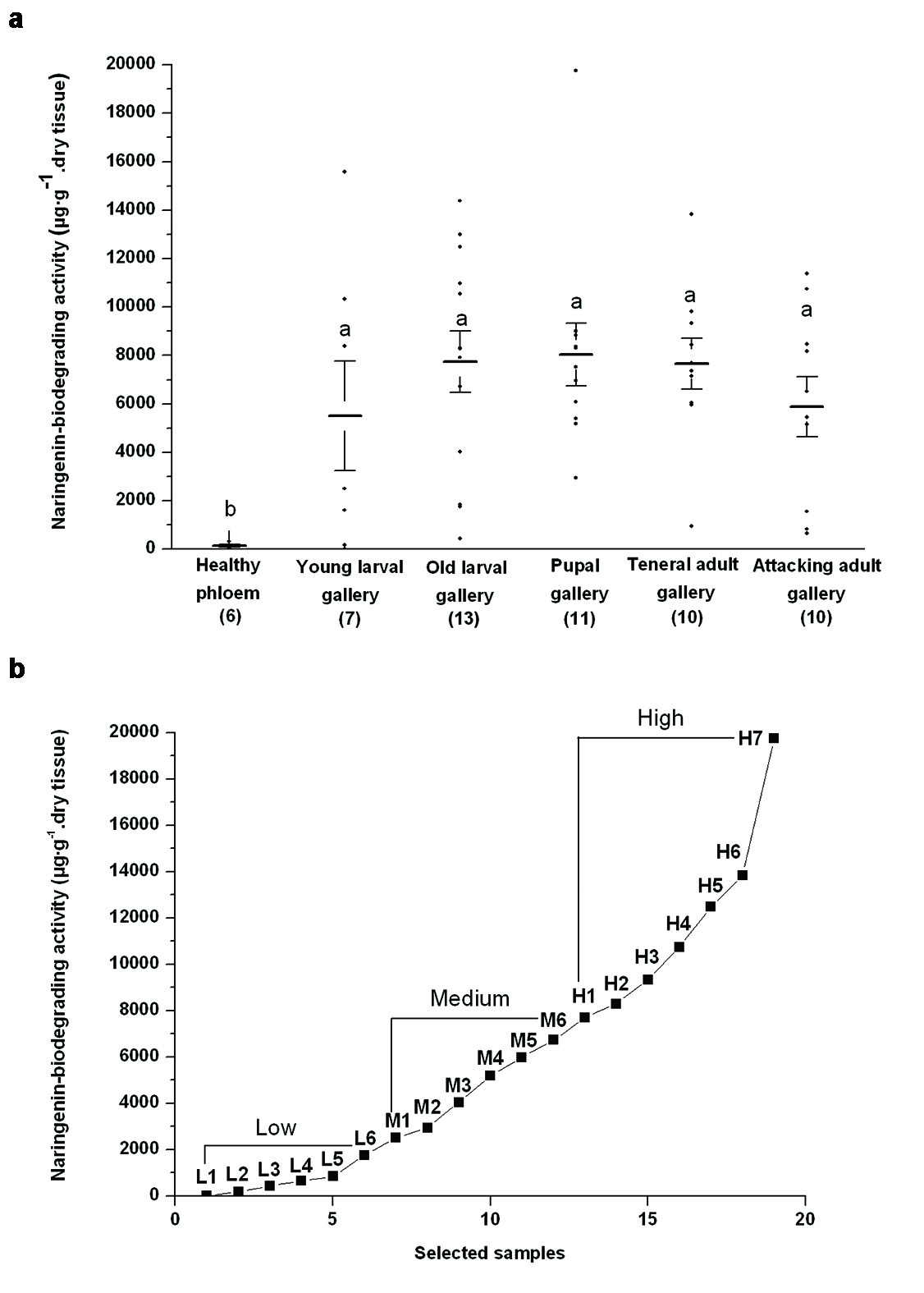
**

**Figure S2.** Rarefaction curves of the 19 samples for bacterial OTUs (**a**) and fungal OTUs (**b**). Red lines represent low-activity group samples; Green lines represent medium-activity group samples; Blue lines represent high-activity group samples. Sample names were corresponded to those shown in Fig. S1b.


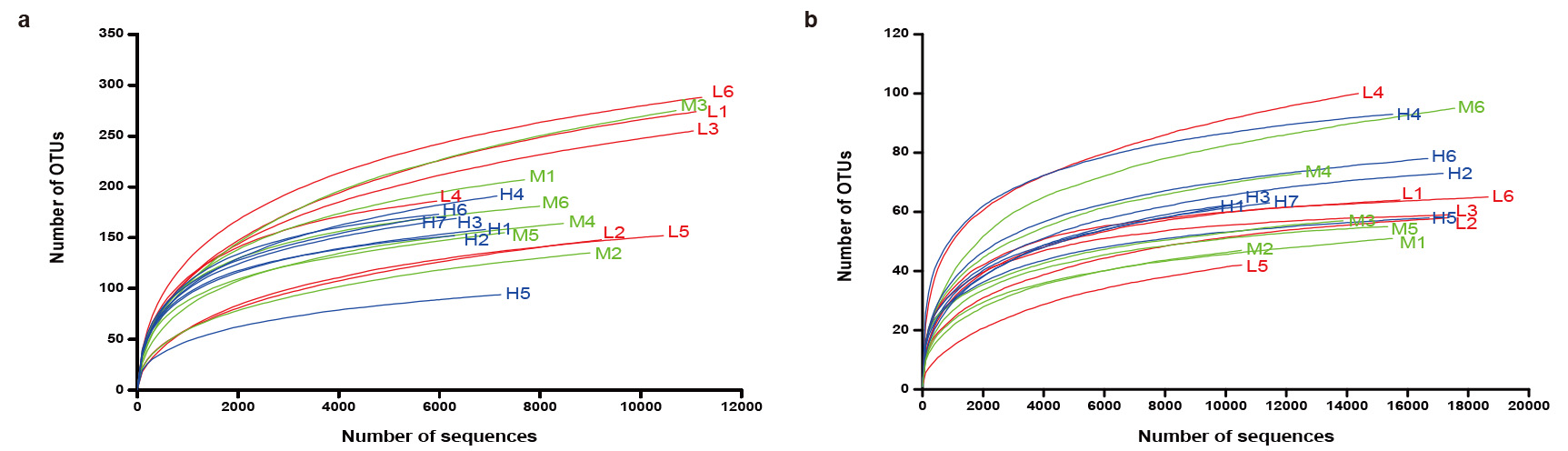


**Figure S3.** Effects of anti-fungal and anti-bacterial treatments on naringenin degradation, under liquid (**a**) and solid (**b**) media condition. NC: nystatin and cycloheximide; SP: streptomycin and penicillin. ** *P* < 0.01.

**
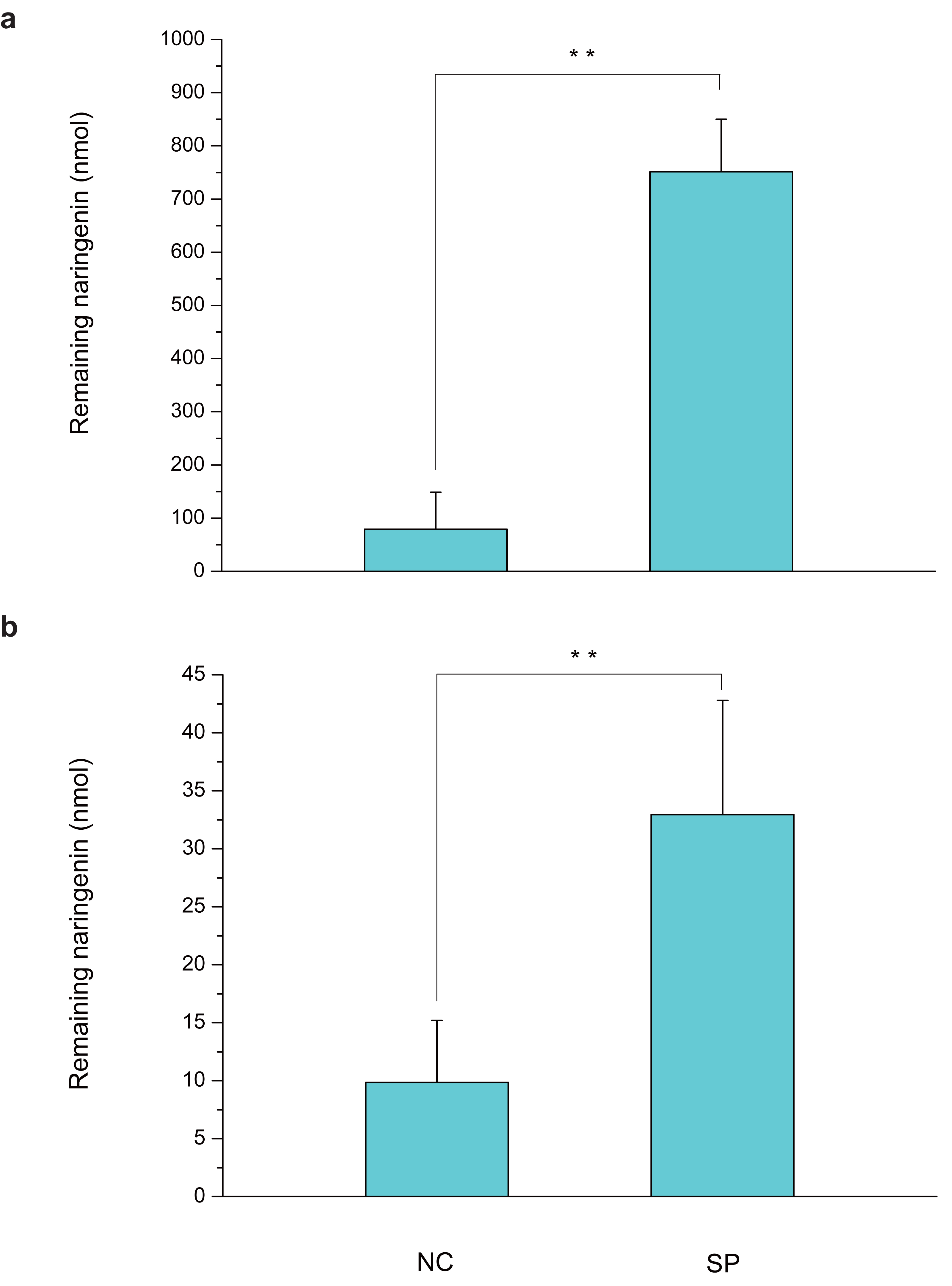
**

**Figure S4.** The rank abundance diagram of the 708 bacterial OTUs (**a**) and 209 fungal OTUs (**b**) identified, plotted as the traditional Whittaker plot. Dots above the red lines mean the ones are > 1% in relative abundance.


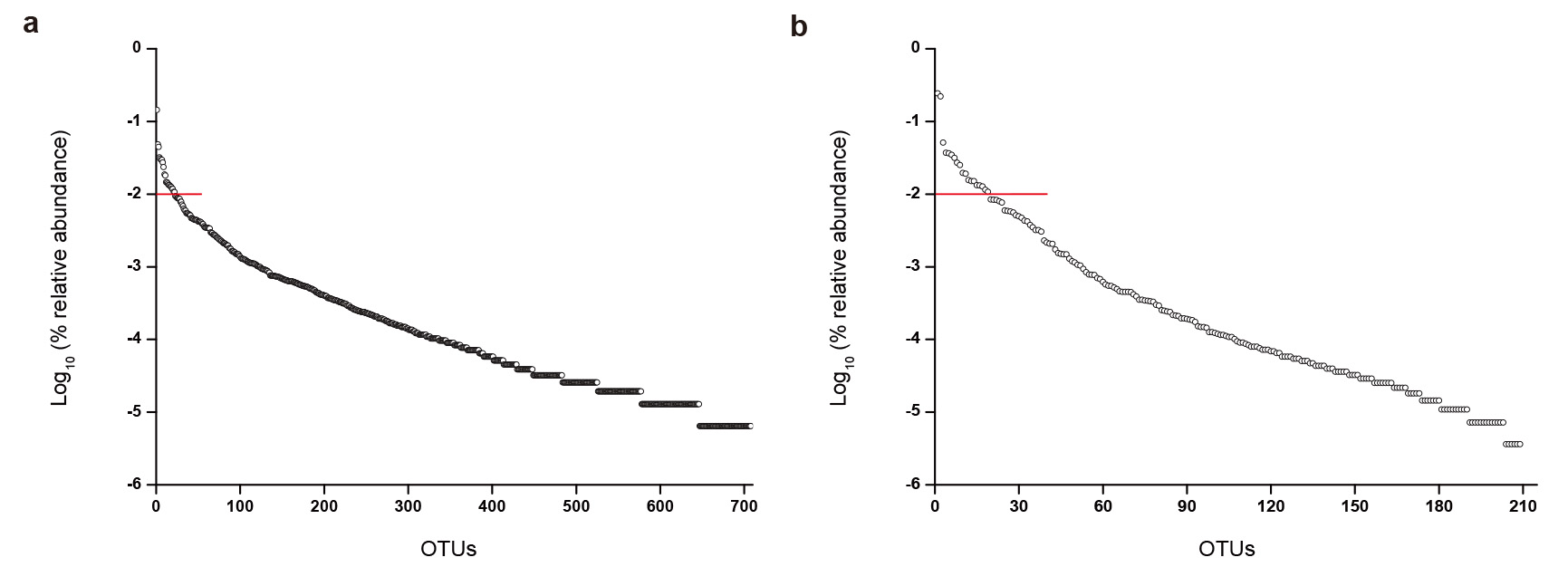


**Figure S5.** Effects of anti-bacterial treatments on naringenin degradation (**a**) and abundance of Gram-negative bacteria (**b**). Different letters on error bars mean significant difference between treatments.

**
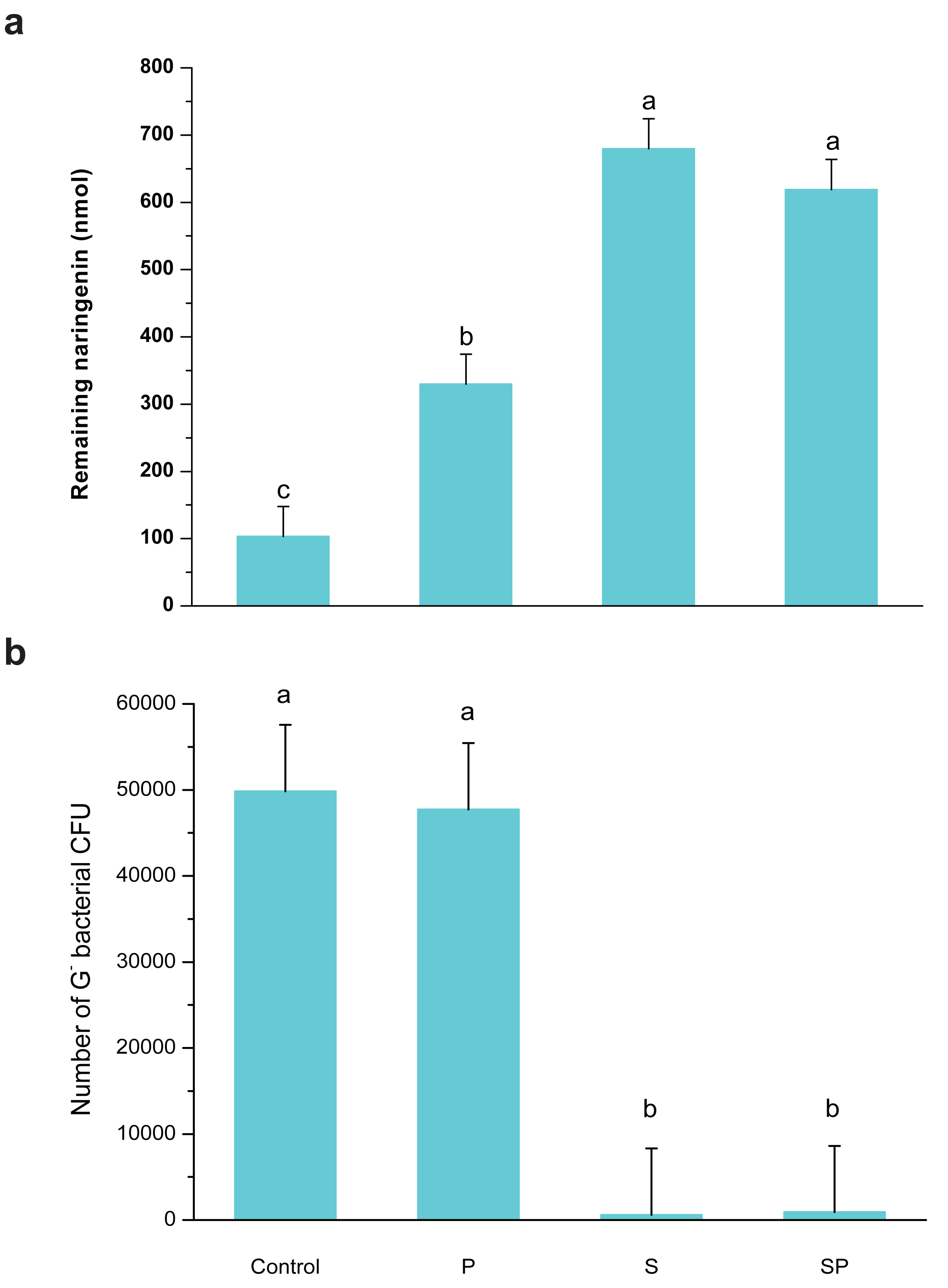
**

**Figure S6.** KEGG pathway annotations for Gram-negative bacteria (**a**) and Gram-positive bacteria (**b**) in RTB gallery microbiota.

**
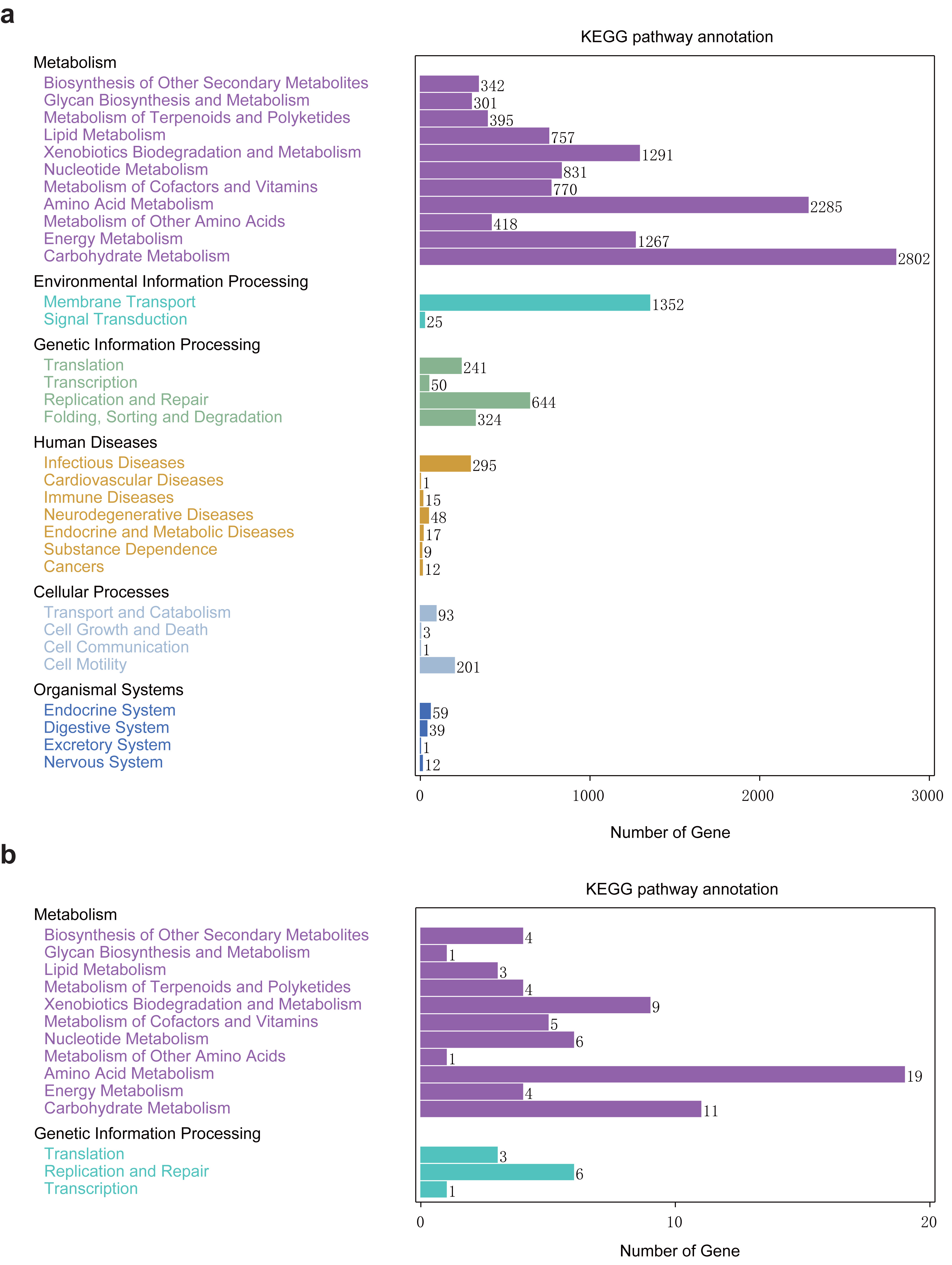
**

**References**

1 Aoki K, Konohana T, Shinke R, Nishira H. Two catechol 1,2-dioxygenases from an aniline-assimilating bacterium, *Frateuria* species ANA-18. Agric Biol Chem. 1984;48:2097-104.

2 Kanaly RA, Harayama S, Watanabe K. *Rhodanobacter* sp. strain BPC1 in a benzo[a]pyrene-mineralizing bacterial consortium. Appl Environ Microbiol. 2002;68:5826-33.

3 Kim JM, Le NT, Chung BS, Park JH, Bae JW, Madsen EL, et al. Influence of soil components on the biodegradation of benzene, toluene, ethylbenzene, and *o*-, *m*-, and *p*-xylenes by the newly isolated bacterium *Pseudoxanthomonas spadix* BD-a59. Appl Environ Microbiol. 2008;74:7313-20.

4 Liu Z, Yang C, Qiao C. Biodegradation of *p*-nitrophenol and 4-chlorophenol by *Stenotrophomonas* sp. FEMS Microbiol Lett. 2007;277:150-6.

5 Leigh MB, Prouzová P, Macková M, Macek T, Nagle DP, Fletcher JS, et al. Polychlorinated biphenyl (PCB)-degrading bacteria associated with trees in a PCB-contaminated site. Appl Environ Microbiol. 2006;72:2331-42.

6 Li A, Qu Y, Zhou J, Gou M. Isolation and characteristics of a novel biphenyl-degrading bacterial strain, *Dyella ginsengisoli* LA-4. J Environ Sci. 2009;21:211-7.

7 Palleroni NJ, Port AM, Chang HK, Zylstra GJ. *Hydrocarboniphaga effusa* gen. nov., sp. nov., a novel member of the *γ*-Proteobacteria active in alkane and aromatic hydrocarbon degradation. Int J Syst Evol Microbiol. 2004;54:1203-07.

8 Nogales J, Canales Á, Jiménez-Barbero J, Serra B, Pingarrón JM, García JL, et al. Unravelling the gallic acid degradation pathway in bacteria: the *gal* cluster from *Pseudomonas putida*. Mol Microbiol. 2011;79:359-74.

9 Adav SS, Chen MY, Lee DJ, Ren NQ. Degradation of phenol by *Acinetobacter* strain isolated from aerobic granules. Chemosphere. 2007;67:1566-72.

10 Di Gioia D, Michelles A, Pierini M, Bogialli S, Fava F, Barberio C. Selection and characterization of aerobic bacteria capable of degrading commercial mixtures of low-ethoxylated nonylphenols. J Appl Microbiol. 2008;104:231-42.

11 Pepi M, Lampariello LR, Altieri R, Esposito A, Perra G, Renzi M, et al. Tannic acid degradation by bacterial strains *Serratia* spp. and *Pantoea* sp. isolated from olive mill waste mixtures. Int Biodeterior Biodegrad. 2010;64:73-80.

12 Li J, Jin Z, Yu B. Isolation and characterization of aniline degradation slightly halophilic bacterium, *Erwinia* sp. Strain HSA 6. Microbiol Res. 2010;165:418-26.

13 De Los Cobos-Vasconcelos D, Santoyo-Tepole F, Juárez-Ramírez C, Ruiz-Ordaz N, Galíndez-Mayer CJJ. Cometabolic degradation of chlorophenols by a strain of *Burkholderia* in fed-batch culture. Enzyme Microb Technol. 2006;40:57-60.

14 Liz JAZE, Jan-Roblero J, Serna JZD, León AVP, Hernández-Rodríguez C. Degradation of polychlorinated biphenyl (PCB) by a consortium obtained from a contaminated soil composed of *Brevibacterium*, *Pandoraea* and *Ochrobactrum*. World J Microbiol Biotechnol. 2009;25:165-70.

15 Louie TM, Webster CM, Xun L. Genetic and biochemical characterization of a 2,4,6-trichlorophenol degradation pathway in *Ralstonia eutropha* JMP134. J Bacteriol. 2002;184:3492-500.

16 Allison N, Turner JE, Wait R. Degradation of homovanillate by a strain of *Variovorax paradoxus* via ring hydroxylation. FEMS Microbiol Lett. 1995;134:213-19.

17 Bae HS, Lee JM, Kim YB, Lee ST. Biodegradation of the mixtures of 4-chlorophenol and phenol by *Comamonas testosteroni* CPW301. Biodegradation. 1996;7:463-9.

18 Juárez-Jiménez B, Manzanera M, Rodelas B, Martínez-Toledo MV, Gonzalez-López J, Crognale Silvia, et al. Metabolic characterization of a strain (BM90) of *Delftia tsuruhatensis* showing highly diversified capacity to degrade low molecular weight phenols. Biodegradation. 2009;21:475-89.

19 Wan N, Gu JD, Yan Y. Degradation of *p*-nitrophenol by *Achromobacter xylosoxidans* Ns isolated from wetland sediment. Int Biodeterior Biodegrad. 2007;59:90-6.

20 Uchihashi K, Misawa T, Takeo M, Negoro S. Mutational analysis of the metabolism of 2,6-naphthalenedisulfonate by *Pigmentiphaga* sp. NDS-2. J Biosci Bioeng. 2003;95:476-82.

21 Hess TF, Silverstein J, Schmidt SK. Effect of glucose on 2,4-dinitrophenol degradation kinetics in sequencing batch reactors. Water Environ Res. 1993;65:73-81.

22 Xu HX, Wu HY, Qiu YP, Shi XQ, He GH, Zhang JF, et al. Degradation of fluoranthene by a newly isolated strain of *Herbaspirillum chlorophenolicum* from activated sludge. Biodegradation. 2011;22:335-45.

23 Tiirola MA, Männistö MK, Puhakka JA, Kulomaa MS. Isolation and Characterization of *Novosphingobium* sp. strain MT1, a dominant polychlorophenol-degrading strain in a groundwater bioremediation system. Appl Environ Microbiol. 2002;68:173-80.

24 Ederer MM, Crawford RL, Herwig RP, Orser CS. PCP degradation is mediated by closely related strains of the genus *Sphingomonas*. Mol Ecol. 1997;6:39-49.

25 Copley SD, Rokicki J, Turner P, Daligault H, Nolan M, Land M. The whole genome sequence of *Sphingobium chlorophenolicum* L-1: insights into the evolution of the pentachlorophenol degradation pathway. Genome Biol Evol. 2012;4:184-98.

26 Teramoto M, Suzuki M, Hatmanti A, Harayama S. The potential of *Cycloclasticus* and *Altererythrobacter* strains for use in bioremediation of petroleum-aromatic-contaminated tropical marine environments. J Biosci Bioeng. 2010;110:48-52.

27 Stapleton RD, Savage DC, Sayler GS, Stacey G. Biodegradation of aromatic hydrocarbons in an extremely acidic environment. Appl Environ Microbiol. 1998;64:4180-4.

28 Hopper W, Mahadevan A. Degradation of catechin by *Bradyrhizobium japonicum*. Biodegradation**.** 1997;8:159-65.

29 Yang CF, Lee CM. Enrichment, isolation, and characterization of 4-chlorophenol-degrading bacterium *Rhizobium* sp. 4-CP-20. Biodegradation. 2008;19:329-36.

30 Li J, Gu JD. Complete degradation of dimethyl isophthalate requires the biochemical cooperation between *Klebsiella oxytoca* Sc and *Methylobacterium mesophilicum* Sr isolated from wetland sediment. Sci Total Environ. 2007;380:181-7.

31 Fetzner S. Bacterial degradation of pyridine, indole, quinoline, and their derivatives under different redox conditions. Appl Microbiol Biotechnol. 1998;49:237-50.

32 Xie S, Sun W, Luo C, Cupples AM. Novel aerobic benzene degrading microorganisms identified in three soils by stable isotope probing. Biodegradation. 2011;22:71-81.

33 Cho YG, Yoon JH, Park YH, Lee ST. Simultaneous degradation of *p*-nitrophenol and phenol by a newly isolated *Nocardioides* sp. J Gen Appl Microbiol. 1998;44:303-9.

34 Häggblom MM, Nohynek LJ, Salkinoja-Salonen MS. Degradation and *O*-methylation of chlorinated phenolic compounds by *Rhodococcus* and *Mycobacterium* strains. Appl Environ Microbiol. 1988;54:3043-52.

35 Kim EJ, Jeon JR, Kim YM, Murugesan K, Chang YS. Mineralization and transformation of monofluorophenols by *Pseudonocardia benzenivorans*. Appl Microbiol Biotechnol. 2010;87:1569-77.

36 Steiert JG, Pignatello JJ, Crawford RL. Degradation of chlorinated phenols by a pentachlorophenol-degrading bacterium. Appl Environ Microbiol. 1987;53:907-10.

37 Li T, Deng XP, Wang JJ, Zhao H, Wang L, Qian K*.* Biodegradation of 3,4-dichloroaniline by a novel *Myroides odoratimimus* strain LWD09 with moderate salinity tolerance. Water Air Soil Pollut. 2012;223:3271-9.

38 Wang D, Lin Y, Du W, Liang J, Ning Y. Optimization and characterization of lignosulfonate biodegradation process by a bacterial strain, *Sphingobacterium* sp. HY-H. Int Biodeterior Biodegrad. 2013;85:365-71.
