# Supplementary figures and images for: Bacterial microbiota protect an invasive bark beetle from a pine defensive compound
Source: Microbiome. 2018 Jul 27;6:132. doi: 10.1186/s40168-018-0518-0 (PMC6064089; doi:10.1186/s40168-018-0518-0)

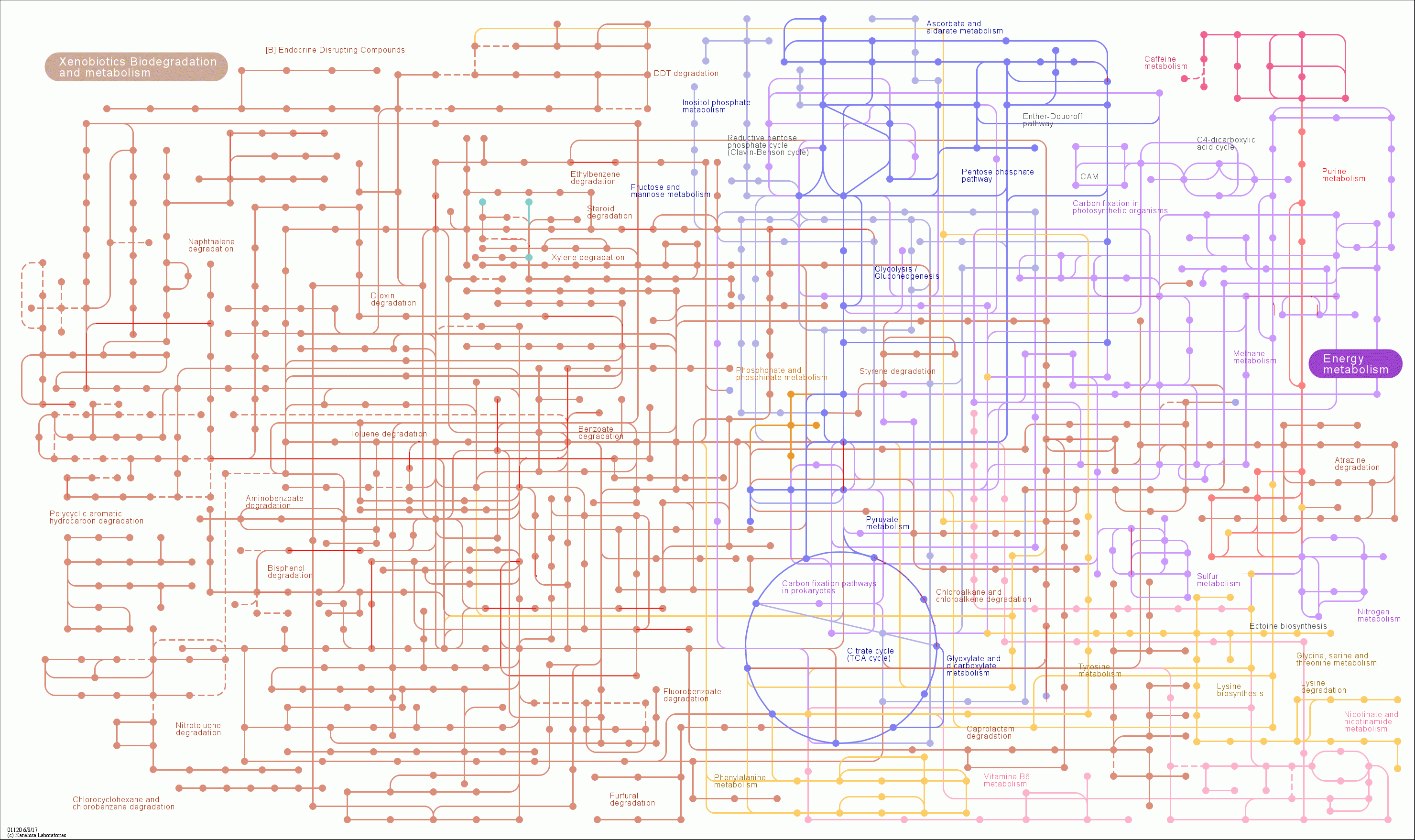

Supplement: Supplementary file 2 — map01120. Projection of genes involved in microbial metabolism in diverse environments on the KEGG pathways. (PNG 276 kb) [file 40168_2018_518_MOESM2_ESM.png]
