# Supplementary material for: Bacterial microbiota protect an invasive bark beetle from a pine defensive compound
Source: Microbiome. 2018 Jul 27;6:132. doi: 10.1186/s40168-018-0518-0 (PMC6064089; doi:10.1186/s40168-018-0518-0)
Supplement: Supplementary file 3 — Gene IDs of KEGG pathways in map01120. (HTML 848 kb) [file 40168_2018_518_MOESM3_ESM.html]

map01120
